# Telling You More Fluently: Effect of the Joint Presentation of Eco-Label Information on Consumers’ Purchase Intention

**DOI:** 10.3390/ijerph192013713

**Published:** 2022-10-21

**Authors:** Xingyuan Wang, Yingying Du, Yun Liu, Shuyang Wang

**Affiliations:** School of Management, Shandong University, Jinan 250100, China

**Keywords:** eco-label, joint presentation of eco-label information, cognitive fluency, spatial contiguity, construal level

## Abstract

An eco-label is an important tool for identifying green products in the marketplace. Most eco-labels, however, present a single icon that is simple and carries limited information, thus creating cognitive barriers for consumers. As a result, eco-labels might not always effectively promote green consumption. Based on dual coding theory and the spatial contiguity effect, this study investigated the effect of the “joint presentation of eco-label information” (JPEI), which adds (functional/emotional) descriptive text to eco-labels, on improving consumers’ cognitive fluency in eco-labels and subsequent purchase intention. We conducted three studies and found that, compared with the “single presentation of eco-label information” (SPEI), JPEI improved the cognitive fluency of consumers with low eco-label knowledge. Furthermore, spatially contiguous JPEI was more effective than spatially partitioned JPEI for consumers with low eco-label knowledge. In addition, we specifically explored the information types of JPEI that were effective for consumers with low eco-label knowledge. Low-construal consumers had higher cognitive fluency and higher purchase intentions under functional JPEI, and high-construal consumers had higher cognitive fluency and higher purchase intentions under emotional JPEI. The results of this study enrich eco-label research and can provide theoretical guidance for marketing practices in eco-labels.

## 1. Introduction

Eco-labels are used on product packaging to convey that a product has green attributes. Although eco-labels are widely used in green marketing, some studies have identified obstacles to their effective application [1]. Because of the simple format of most eco-labels, the limited information they convey [2], and the varied types of eco-label icons (the 2021 Eco-Label Index identified 455 different labels in 199 countries; http://www.ecolabelindex.com/, accessed on 30 June 2022), consumers often have difficulty recognizing and understanding eco-label information [3,4]. One eye-tracking study, for example, found that participants recognized and understood only two of 110 eco-labels [5]. It has also been found that eco-labels are mainly effective for consumers who already possess environmental awareness [6] and less effective for those who do not. Eco-labels are only effective when consumers can readily understand their meanings [2]. However, many consumers have cognitive difficulties related to eco-labels. A previous study in China also concluded that the eco-label system is very complex for consumers and that there is a need to improve consumers’ understanding of eco-labels [7].

Recent research on eco-labels has focused on their effects on consumers’ attitudes, purchase intentions, purchase behaviors, and willingness to pay a premium for green products [3,8,9,10,11]. Such effects are mainly related to the visibility of eco-label designs [12,13], eco-label certification sources [14], eco-label formats [15], consumers’ knowledge of eco-labels or ecology [10,16], product quality inferences and product evaluation influenced by eco-labels [17,18], and consumers’ attitudes toward eco-labels [19,20]. While some studies have acknowledged the difficulties some consumers face in understanding eco-labels [3,4,5]—that is, their cognitive fluency in eco-labels—there remains a gap in the literature regarding how to address the problem [12]. To address the cognitive problem of eco-labels, we will mainly discuss the following aspects.

First, we take insights from dual coding theory (DCT), which proposes that verbal (e.g., text) and nonverbal (e.g., icon or image) codes corresponding to the same object can have additive effects on cognition [21]. Many eco-labels are presented as single icons and are nonverbal codes, which was conceptualized as “single presentation of eco-label information” (SPEI) in this study. We propose the eco-label format of “joint presentation of eco-label information” (JPEI), which adds descriptive text as verbal codes to single-icon eco-labels to improve consumers’ cognition and understanding of eco-labels. Regarding the information types to add for the descriptive text, we referred to the classification of information types in green advertising and divided the descriptive text into functional and emotional types [22], thus forming emotional JPEI and functional JPEI. However, studies on DCT have found that the effect of dual coding is related to individual characteristics, such as individuals’ prior knowledge [23]. Based on this, we took consumers’ eco-label knowledge as a moderator and explored whether JPEI was more effective than SPEI for improving consumers’ cognitive fluency in eco-labels.

Second, if, as predicted, JPEI is indeed more effective than SPEI for consumers’ cognition, how should JPEI be spatially located? In the extending research on DCT, scholars have proposed that individuals can cognize better when corresponding verbal and non-verbal codes are presented close to each other rather than far apart, which is known as the “spatial contiguity effect” [24]. This effect is also related to individuals’ prior knowledge [25]. Thus, we used consumers’ eco-label knowledge as a moderator to explore how the spatial distance of JPEI (spatially contiguous vs. spatially partitioned) can improve cognitive fluency in eco-labels. In addition, we explored whether increased cognitive fluency leads to higher purchase intentions.

Third, if JPEI is effective for consumers with a specific eco-label knowledge level, then we need to consider when to use functional JPEI or emotional JPEI. To examine this, we referred to construal level theory for insights into the effectiveness of functional vs. emotional JPEI. Specifically, we explored how functional JPEI and emotional JPEI can be matched to consumers’ construal level to generate higher cognitive fluency and purchase intentions.

This work enriches research on green consumption, especially eco-labels. We proposed the concept of “joint presentation of eco-label information” (JPEI), and examined the effects of spatial distance and information types in JPEI, which fills the research gap of eco-labels in cognitive perspective. Additionally, we constructed a framework in which eco-label presentation influences consumers’ purchase intentions. Our research also provides guidance for the future use of eco-labels.

## 2. Literature Review and Hypothesis Development

Ecolabels are symbols designed to identify and distinguish products that have a positive environmental effect [1]. While green products typically use eco-labels to communicate green attributes, there are barriers to consumers’ cognition [26]. This is mainly because there are many types of eco-label icons, some of them are very simple in format, and they convey limited information [2,14,17]. Eco-labels do not effectively transmit green information between buyers and sellers [27]. Carrero et al. [28] and Taufique et al. [13] noted that consumers have difficulty understanding and recognizing eco-labels, especially those who have low eco-label knowledge [29], making it difficult for eco-labels to serve their purpose in the purchasing process [30]. Therefore, how to make it easier for consumers to understand and recognize eco-labels is an important research topic. Cognitive fluency reflects the ease with which the meaning of information enters one’s mind [31]. This reflects the ease of higher-level processing, such as understanding the meaning of stimuli [32], which is often associated with purchase intentions [33]. Thus, our study explored whether JPEI can influence product purchase intentions by improving consumers’ cognitive fluency of eco-labels.

While previous studies have recognized that eco-label formatting affects purchase decisions, there is little research in this area [12]. An existing study found that icon-based eco-labels attracted more visual attention than text-based ones and that visual attention to icon-based eco-labels increased consumer bids [12]. In research on other types of labels, Jaud and Melnyk [34] showed that wine labels combining text with matching images were better than text-only labels and labels where images and text do not match. No studies, however, have specifically examined the effect of eco-labels with a combined format on consumers’ cognition. Some research in the field of advertising has explored the effect of combinations of different information formats. Kim et al. and Sahin et al. found that combinations of logo–text or image–text designs were better than single-format designs [35,36]. Furthermore, some studies have suggested that when people combine images and textual information, their comprehension behavior is largely text driven; that is, people first use the text to construct an initial representation and then combine it with information from the images [37,38]. This suggests that descriptive eco-label text can facilitate consumers’ understanding. Rihn et al. [12] also proposed that textual eco-labels may be useful for educating consumers about less familiar logos. In the next section, we will specifically elaborate on how JPEI enhances consumer purchase intentions through cognitive fluency.

### 2.1. Eco-Label Information Presentation, Consumers’ Eco-Label Knowledge and Cognitive Fluency

Based on DCT, when individuals apply verbal and non-verbal coding in information processing, the joint processing of the two can enable more efficient interactions between them and improve individuals’ learning and cognition [39]. Research on DCT has also suggested that nonverbal coding is more detrimental to cognitive learning than verbal coding [40]. When nonverbal coding is difficult to understand, the corresponding verbal coding can form a reference link with the nonverbal coding to help improve cognition [39]. However, nonverbal coding is cognitively efficient when individuals have a priori knowledge about nonverbal coding [41]. We therefore introduced consumers’ eco-label knowledge as a moderator.

We defined consumers’ eco-label knowledge as the level of information about a particular eco-label that consumers perceived to be stored in their memory [42]. Consumers’ knowledge can influence consumers’ perceptions of their ability to process relevant information [43]. Consumers with high and low knowledge differ in the presentation structure of their target. Specifically, those with low knowledge have a fragmented and shallow cognitive schema and do not have well-developed criteria for making decisions based on existing knowledge [44,45]; thus, they need to search for more external information to support their understanding [46]. Consumers with high knowledge are more likely to process information using prior knowledge and decision criteria readily available to them and are therefore less likely to rely on external heuristic cues [47].

**Figure 1:**
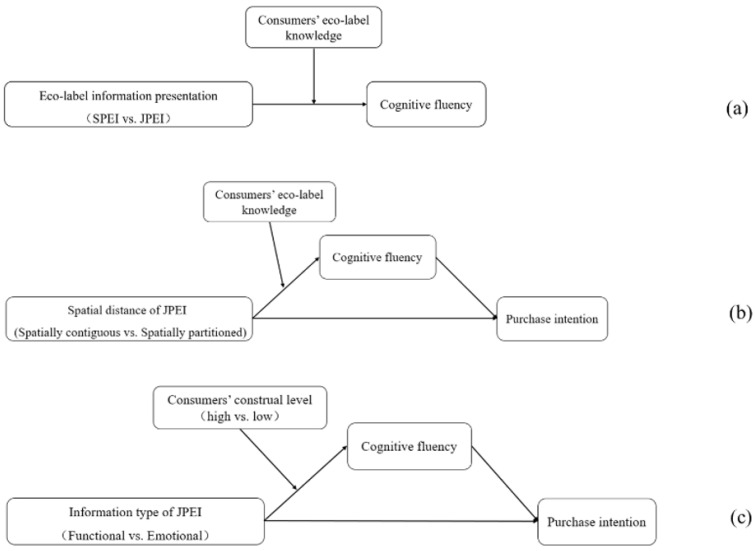
Conceptual model (Note: (**a**) shows H1a and H1b; (**b**) shows H2a and H2b; (**c**) shows H3a and H3b).

In this study, the eco-label icon is nonverbal coding, and the eco-label descriptive text is verbal coding. Consumers with low eco-label knowledge have difficulty automatically activating eco-label processing, but consumers with high eco-label knowledge can [48]. We infer that JPEI can make consumers with low eco-label knowledge more fluent in their eco-label cognition because it provides more information [27] and allows for the joint processing of dual coding (i.e., an eco-label icon and its descriptive text). However, consumers with high eco-label knowledge are more likely to make purchasing decisions by simply identifying the eco-label icon [10] without relying on additional descriptive text. Thus, we propose the following interaction effects (see Figure 1a):

**H1a.**  *For consumers with low eco-label knowledge, JPEI can increase their cognitive fluency in eco-labels compared to SPEI.*

**H1b.**  *For consumers with high eco-label knowledge, there are no significant differences in the effects of SPEI and JPEI on their cognitive fluency in eco-labels.*

### 2.2. Spatial Distance of JPEI, Consumers’ Eco-Label Knowledge, Cognitive Fluency and Purchase Intentions

In this study, spatial distance refers to an interval of geometric distance between design elements [49]; it can be either spatially contiguous or spatially partitioned [50]. Research on multimedia learning suggests the spatial distance between image and text affects cognitive or learning effects [51], and proposes the theory of “spatial contiguity effect” [52]. When images and text are both needed to understand a concept, spatial contiguity between them can reduce the time individuals spend searching for information, and they can retain more information in their short-term memory [53], which better facilitates learning [54]. When information is spatially partitioned, individuals become prone to attentional separation [50], in which case their cognitive load increases owing to the need to mentally combine different sources of information [55], which can result in reduced cognitive effects [52]. However, Mayer proposed that the spatial contiguity effect should consider the prior knowledge of individuals [25]. Spatial distance has less effect on individuals with higher prior knowledge [56]. This is because the eye movement paths of individuals with high prior knowledge are characterized by higher saccade lengths [57]. Thus, individuals with high prior knowledge are better able to visually and cognitively connect spatially distant elements to fully comprehend the information [53].

For consumers with low eco-label knowledge, spatially contiguous JPEI can facilitate consumers’ visual search for information, and a good referential link between an eco-label and descriptive text will also improve cognitive fluency [56]. When JPEI is spatially partitioned, consumers need to process information from different locations, thus experiencing split attention and a greater cognitive load [52], which results in relatively low cognitive fluency. However, consumers with high eco-label knowledge rely less on other information cues and more on their prior knowledge to process eco-labels [46]. Furthermore, even if consumers with high eco-label knowledge see the descriptive text, they can quickly link the eco-label and the text cognitively to complete their information processing [53]. Based on this, we predict that spatial distance will not have a significant effect on their cognitive fluency. Previous studies have found that cognitive fluency can positively influence consumers’ purchase intentions [33]. Separately, Sigurdsson et al. also found that having an understanding of eco-labels can have a positive effect on purchase intentions [3]. Thus, we propose the following interaction effects (see Figure 1b):

**H2a.**  *For consumers with low eco-label knowledge, spatially contiguous JPEI will enhance their cognitive fluency in eco-labels compared to spatially partitioned JPEI and then lead to higher purchase intentions.*

**H2b.**  *For consumers with high eco-label knowledge, there will be no significant difference in the effect of spatially contiguous JPEI and spatially partitioned JPEI on cognitive fluency and, subsequently, purchase intentions.*

### 2.3. Information Type of JPEI, Consumers’ Construal Level, Cognitive Fluency and Purchase Intentions

Construal level refers to the extent to which an individual is in an abstract mind-set (i.e., focused on objects’ superordinate and central features) versus a concrete mind-set (i.e., focused on objects’ subordinate and specific features) [58]. The theory proposes that one’s construal level affects the processes of receiving, processing, and responding to information, as well as persuasion [59]. When the information type matches individuals’ construal level, the matching information will produce a more fluent encoding process, which will positively affect individuals’ purchase decisions [60,61]. At a low construal level, people typically focus on more specific information from a detailed perspective; at a high construal level, people focus on more abstract information from a central, essential perspective [62]. Some research on branding and advertising has shown that consumers with a high construal level prefer emotional information, while those with a low construal level prefer functional information [62,63]. We likewise believe that consumers’ construal level will be associated with the effectiveness of the information type in eco-labels.

Functional JPEI provided practical information about eco-labels’ environmental attributes [64], while emotional JPEI made emotional appeals about eco-labels to promote pro-environmental behavior [65]. Functional JPEI provides more concrete, realistic, and detailed information, which is more in line with the cognitive habits of low-construal consumers; emotional JPEI provides more abstract information and expresses a good vision, and is more in line with the cognitive habits of high-construal consumers [63]. Thus, functional JPEI should lead to more fluent eco-label cognition for low-construal consumers, and emotional JPEI should lead to more fluent eco-label cognition for high-construal consumers. In the above analysis, we have proposed that fluent cognition of eco-label information will positively affect consumers’ purchase intentions [3,33]. Thus, we propose the following interaction effects (see Figure 1c):

**H3a.**  *For low-construal consumers, functional JPEI will lead to higher cognitive fluency than emotional JPEI, which in turn will lead to higher purchase intentions.*

**H3b.**  *For high-construal consumers, emotional JPEI will lead to higher cognitive fluency than functional JPEI, which in turn will lead to higher purchase intentions.*

Figure 1 depicts the conceptual model based on all of the above hypotheses.

## 3. Overview of Studies

Three experiments were designed to test the hypotheses. In Study 1, a two-piece (eco-label information presentation: SPEI vs. JPEI) × continuous (consumers’ eco-label knowledge) between-subjects design was conducted to test whether JPEI could improve consumers’ cognitive fluency in eco-labels, using consumers’ eco-label knowledge as the moderator. Based on Study 1, Study 2 also used consumers’ eco-label knowledge as the moderator to test whether spatial contiguous JPEI would more effectively improve cognitive fluency compared to spatial partitioned JPEI and whether that improvement would positively affect purchase intentions. A two-piece (spatial distance of JPEI: spatially contiguous vs. spatially partitioned) × continuous (consumers’ eco-label knowledge) between-subjects design was adopted in Study 2. Study 3 used consumers’ construal level as the moderator to test which information type of JPEI (functional vs. emotional) is more effective in increasing cognitive fluency among consumers with low eco-label knowledge and whether it would subsequently increase purchase intentions. A two-piece (JPEI information type: functional vs. emotional) × two-piece (consumers’ construal level: high vs. low) between-subjects design was used. The experimental stimulus used in Studies 1 and 2 was the FSC eco-label, and Study 3 used the “Euro-leaf” eco-label. We also performed a pre-test to justify the selection of stimuli in the experiments.

## 4. Study 1

Study 1 tested whether JPEI could improve participants’ cognitive fluency in eco-labels more than SPEI under the boundary condition of participants’ eco-label knowledge.

### 4.1. Method

#### 4.1.1. Participants and Design

A total of 240 participants were randomly recruited from Credamo (www.credamo.com, accessed on 30 June 2022) and offered a reward (see Supplementary Material E, in Supplementary Materials, for demographic profiles of participants). Credamo is considered to have significant reliability [66], and a number of studies using Credamo have been published in leading journals (e.g., [67,68]. We adopted a 2 (eco-label information presentation: SPEI vs. JPEI) × continuous (consumers’ eco-label knowledge) between-subjects design. To increase authenticity, we selected a commonly used tissue as the stimulus and used the virtual brand “ECO” tissue to exclude interference from the brand factor. We selected “FSC” certification as the eco-label, because Tan et al. found that 21% of participants knew the FSC eco-label in Chongqing shopping for wood flooring products [69]. Currently, more consumers are familiar with the FSC eco-label. We conducted a pre-test to justify the eco-label selection. Regarding the added descriptive text, as mentioned previously, it could be either functional or emotional, and we tested the design in the pre-test.

We recruited 120 participants for the pre-test (see Supplementary Material E for demographic profiles of participants). First, the participants were asked to look at the image of the FSC eco-label and then answer the question, “Do you recognize the FSC eco-label?” (1 = yes, 2 = no). The results showed that approximately half (56.7%) of the participants recognized the FSC eco-label, indicating that the stimulus was reasonably selected.

Second, we performed a manipulation test on functional (*n* = 60) and emotional (*n* = 60) descriptive text. For functional descriptive text, following Matthes et al. [22] regarding functional claims, it was “This product has been certified by ‘FSC Forest Stewardship Council’ certification. Made from 100% FSC-certified, well-managed forests. Meets the FSC certification criteria for environmental suitability and community benefit.” For emotional descriptive text, again following Matthes et al. [22], the text included emotional appeals as well as exclamation points at the end of sentences, which help mobilize emotions [70,71]. Referring to Aagerup et al. [72], emotional eco-label descriptive text was “Want a better environment and a more sustainable society? Then choose products certified by the ‘FSC Forest Stewardship Council’! Keep forests alive forever and give future generations a better future!”. After presenting the materials to the participants, the participants answered the question, “Does the descriptive text reflect more of a specific environmental function or more of the emotional appeal of the FSC eco-label?” (1 = more specific environmental function; 7 = more emotional appeal) [72]. The results indicated that our manipulation of the descriptive text was successful (M _functional_ = 3.43, SD = 2.070; M _emotional_ = 5.32, SD = 1.546; t (59) = −5.506, *p* < 0.001).

#### 4.1.2. Procedure

We first measured participants’ FSC eco-label knowledge after they viewed the FSC eco-label image. Chang’s mature scale of product knowledge was adapted [73], which included four items (α = 0.913). Then, participants were asked to look at an image of ECO tissue packaging (see Supplementary Material A). For the SPEI group, the packaging had an FSC eco-label. For the JPEI group, the FSC eco-label was accompanied by the same functional descriptive text as in the pre-test (we also tested the emotional descriptive text; see Supplementary Material A: Follow-up Study).

After presenting the materials to the participants, we used the mature scale of Lee and Aaker to measure cognitive fluency [74], which included three items (α = 0.872) (see Supplementary Material D). In addition, participants’ environmental attitudes might have affected the results; so, we measured the participants’ environmental concerns, also using mature four-item scales [22] (α = 0.749). A seven-point Likert scale was used for all items, with 1 indicating “strongly disagree” and 7 indicating “strongly agree.” Finally, demographic variables such as gender and age were measured.

### 4.2. Results

**Figure 2:**
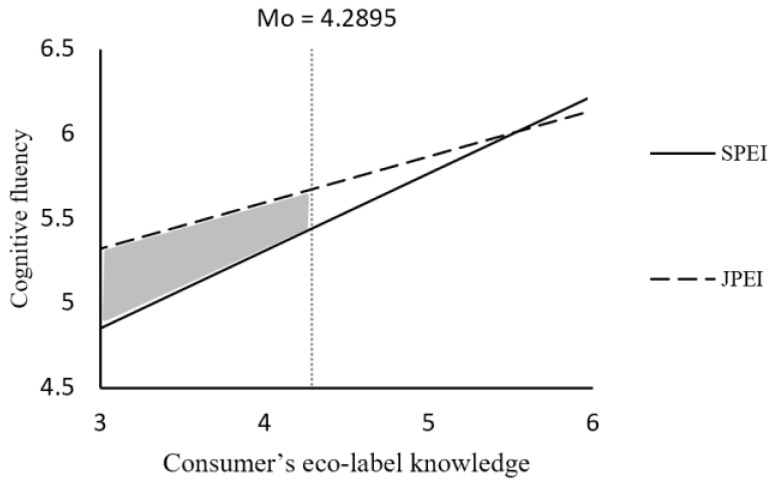
The interactive effect of eco-label information presentation and consumer’s eco-label knowledge (Study 1). Note: the shaded area is the Johnson–Neyman significant area.

There were no significant differences in environmental concern (M _SPEI_ = 5.633, SD = 1.010; M _JPEI_ = 5.700, SD = 0.874; t (238) = −0.547, *p* > 0.05), indicating that it did not affect our results. Eco-label information presentation is a categorical variable (SPEI = 0; JPEI = 1), and consumers’ eco-label knowledge is a continuous variable; thus, we used SPSS 25.0 PROCESS 3.3 (bootstrapping = 5000, 95% CI, Model 1) to test for interaction effects [75]. The results showed that both eco-label information presentation (b = 1.027, SE = 0.352, t = 2.918, *p* < 0.01) and consumers’ eco-label knowledge (b = 0.642, SE = 0.126, t = 5.081, *p* < 0.001) had a significant effect on cognitive fluency. The interaction between eco-label information presentation and consumers’ eco-label knowledge on cognitive fluency is also significant (b = −0.185, SE = 0.078, t = −2.364, *p* < 0.05). We further analyzed the moderating effect using the Johnson–Neyman technique [76]. Supporting H1a and H1b, the results showed that eco-label information presentation had a significant positive effect on cognitive fluency when consumers’ eco-label knowledge was equal to or lower than 4.2895 (*p* = 0.002 to 0.05); when consumers’ eco-label knowledge was higher than 4.2895, the effect of eco-label information presentation on cognitive fluency was not significant (*p* = 0.05 to 0.952, B _JN_ = 4.2895 = 0.233, SE = 0.118) (see Figure 2).

### 4.3. Discussion

The results of Study 1 demonstrated that adding descriptive text to an eco-label (i.e., JPEI) improved the cognitive fluency of participants with low eco-label knowledge. However, for those with high eco-label knowledge, there was no difference between the presence or absence of descriptive text. To enhance the robustness of our results, we conducted another study. In the follow-up to Study 1 (see Supplementary Material A), we used a design similar to that of Study 1 but with a different information type for JPEI (the descriptive text was emotional). The results also supported H1a and H1b.

While this can suggest ways to improve eco-label cognition, enhancing the efficacy of descriptive eco-label text still needs investigation. Specifically, does the spatial distance between the eco-label and text affect cognition? What type of information should be used to make descriptive text more effective for those with low eco-label knowledge? Further, will enhanced cognitive fluency in eco-labels increase participants’ willingness to purchase? These questions were addressed in the two studies that follow.

## 5. Study 2

Study 2 used consumers’ eco-label knowledge as the moderator and investigated the effect of spatial distance on cognitive fluency. Specifically, we studied the differential effects of spatially contiguous and spatially partitioned JPEI on participants’ cognitive fluency and whether such differences in cognitive fluency led to differences in purchase intentions.

### 5.1. Method

#### 5.1.1. Participants and Design

Participants were randomly recruited from Credamo and offered a reward. A 2 (spatial distance of JPEI: spatially contiguous vs. spatially partitioned) × continuous (consumers’ eco-label knowledge) between-subjects design was adopted and 206 participants were recruited (see Supplementary Material E for demographic profiles of participants).

#### 5.1.2. Procedure

The stimuli were similar to those in Study 1, still using the virtual brand ECO tissue and the FSC eco-label. The same scale as in Study 1 was used to measure participants’ knowledge of the FSC eco-label (α = 0.939). In the spatially contiguous JPEI group, the FSC eco-label was placed together with the functional descriptive text, both in the upper right-hand corner of the tissue packaging. In the spatially partitioned JPEI group, the FSC eco-label and functional descriptive text were separated, with the FSC eco-label in the lower left-hand corner and the text in the upper right-hand corner (see Supplementary Material B). We verified the spatial distance manipulation by asking for the distance between the FSC eco-label image and the descriptive text (1 = very close, 7 = very far away). The participants responded to the mature purchase intention scale adapted from Dodds et al. [77] (α = 0.798). Then, cognitive fluency (α = 0.914) and environmental concern (α = 0.636) were measured in the same way as in Study 1 (see Supplementary Material D). All were measured on a seven-point Likert scale, with 1 indicating “strongly disagree” and 7 indicating “strongly agree.” Finally, demographic variables were measured.

### 5.2. Results

#### 5.2.1. Manipulation Test

Participants perceived the spatially partitioned group as more distant than the spatially contiguous group (M _partitioned_ = 5.30, SD = 1.237; M _contiguous_ = 3.70, SD = 1.520; t (204) = 8.277, *p* < 0.01). There were no significant differences in environmental concern between the two groups (M _partitioned_ = 5.842, SD = 0.774; M _contiguous_ = 5.921, SD = 0.634; t (204) = −0.812, *p* > 0.05).

#### 5.2.2. Cognitive Fluency

**Figure 3:**
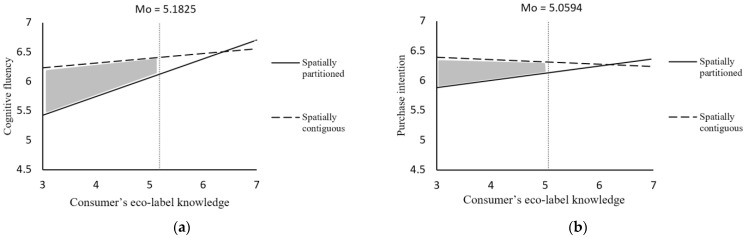
The interactive effect of JPEI spatial distance and consumers’ eco-label knowledge (Study 2). Note: (**a**) shows the interaction effect with cognitive fluency as the dependent variable; (**b**) shows the interaction effect with purchase intention as the dependent variable. The shaded area is the Johnson–Neyman significant area.

Spatial distance is a categorical variable (spatially partitioned = 0; spatially contiguous = 1), and consumers’ eco-label knowledge is a continuous variable; so, we used SPSS 25.0 PROCESS 3.3 (bootstrapping = 5000, 95% CI, Model 1) to test for interaction effects [75]. The results showed that both spatial distance (b = 1.522, SE = 0.378, t = 4.023, *p* < 0.001) and consumers’ eco-label knowledge (b = 0.559, SE = 0.055, t = 10.104, *p* < 0.001) had a significant effect on cognitive fluency. The interaction effect between spatial distance and consumers’ eco-label knowledge on cognitive fluency was also significant (b = −0.239, SE = 0.080, t = −2.967, *p* < 0.01). The Johnson–Neyman technique [76] showed that spatial distance had a significant positive effect on cognitive fluency when consumers’ eco-label knowledge was equal to or lower than 5.1825 (*p* = 0 to 0.05). When consumers’ eco-label knowledge was higher than 5.1825, the effect of spatial distance on cognitive fluency was not significant (*p* = 0.05 to 0.924, B _JN_ = 5.1825 = 0.285, SE = 0.145) (see Figure 3a).

#### 5.2.3. Purchase Intention

The same method was used, and the results showed that both spatial distance (b = 0.994, SE = 0.253, t = 3.936, *p* < 0.001) and consumers’ eco-label knowledge (b = 0.281, SE = 0.037, t = 7.604, *p* < 0.001) had a significant effect on purchase intentions. The interaction effect between spatial distance and consumers’ eco-label knowledge on purchase intentions was also significant (b = −0.160, SE = 0.054, t = −2.979, *p* < 0.01). The Johnson–Neyman [76] results showed that spatial distance had a significant positive effect on purchase intentions when consumers’ eco-label knowledge was equal to or lower than 5.0594 (*p* = 0 to 0.05); when consumers’ eco-label knowledge was higher than 5.0594, the effect of spatial distance on purchase intentions was not significant (*p* = 0.05 to 0.957, B _JN_ =5.0594 = 0.185, SE = 0.094) (see Figure 3b).

#### 5.2.4. Moderated Mediation

**Figure 4:**
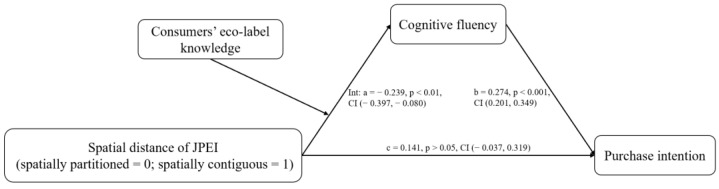
Results for the moderated mediation effect (Study 2).

We used PROCESS 3.3 in SPSS 25.0 to check whether cognitive fluency played a mediating role [75]. Bootstrapping analysis (samples = 5000, 95% CI, Model 7) showed that the mediation of cognitive fluency was significant (indirect effect = −0.066, SE = 0.030, 95% CI = −0.130 to −0.013, excluding 0). Specifically, when participants had low eco-label knowledge, the spatial distance of JPEI had a significant effect on purchase intentions through cognitive fluency (indirect effect = 0.236, SE = 0.085, 95% CI = 0.084 to 0.418, excluding 0); when participants had high eco-label knowledge, the spatial distance of JPEI had a nonsignificant effect on purchase intentions through cognitive fluency (indirect effect = 0.021, SE = 0.032, 95% CI = −0.042 to 0.086, including 0), thus supporting H2a and H2b (see Figure 4).

### 5.3. Discussion

Study 2 demonstrated that when participants had low eco-label knowledge, spatially contiguous JPEI led to higher cognitive fluency and purchase intentions. When participants had high eco-label knowledge, spatially contiguous JPEI and spatially partitioned JPEI showed no significant differences. In addition, we verified it again using the emotional descriptive text and reached the same conclusion (see Supplementary Material B: Study 2 Follow-up A).

In this study, we placed the eco-label image on the lower left-hand corner of the package and the descriptive text on the upper right-hand corner of the package, but the observed effect may be explained by the specific location of the information rather than by the spatial distance. Thus, we conducted two additional studies, which made some changes in spatially partitioned JPEI group. One study placed both the eco-label and the descriptive text at the top of the package but separated them in terms of position. The other study placed the eco-label on the top right-hand corner of the package and the descriptive text on the bottom left-hand corner of the package. The results were consistent with those of Study 2 (see Supplementary Material B: Study 2 Follow-up B and C).

The first two studies revealed that for consumers with low eco-label knowledge, the eco-label should not only be accompanied by descriptive text but that they should also be placed in close proximity to each other. We also showed that both functional and emotional descriptive text can improve cognition for consumers with low eco-label knowledge. In the next study, we explored when to use functional descriptive text or emotional descriptive text.

## 6. Study 3

Study 3 tested the interaction effect between the JPEI information type and consumers’ construal level on cognitive fluency among consumers with low eco-label knowledge. It also tested how changes in cognitive fluency lead to changes in purchase intentions.

### 6.1. Method

#### 6.1.1. Participants and Design

A total of 314 participants were randomly recruited from Credamo and were offered a reward (see Supplementary Material E for demographic profiles of participants). A 2-piece (JPEI information type: functional vs. emotional) × 2-piece (consumers’ construal level: high vs. low) between-subjects design was used. The product was the virtual brand “GRE” oatmeal, which was common in the participants’ daily lives. The eco-label was the “Euro-leaf” organic certification, which has been mandatory in Europe since 2010. A survey in China found that only 16.5% of participants recognized it [78]. We therefore used the Euro-leaf eco-label as the stimulus to better illustrate the effect of JPEI on increasing participants’ cognitive fluency among low eco-label knowledge consumers. We also conducted a pre-test to investigate cognition of the eco-label in China. Construal level manipulation was also tested in the pre-test.

We recruited 120 participants for the pre-test (see Supplementary Material E for demographic profiles of participants). First, the participants were asked to look at the image of the Euro-leaf eco-label and then answer the question, “Do you recognize the eco-label” (1 = yes, 2 = no). The results showed that 18.3% of the participants recognized the eco-label, indicating that cognition of the eco-label is low in China and that most consumers had low knowledge of the eco-label.

Second, we primed the participants’ construal level, using the method proposed by Freitas et al. [79]. “Why” is generally associated with a high construal level, and “how” is generally associated with a low construal level. Four consecutive “why” (*n* = 60) and “how” (*n* = 60) questions were asked on the topic of “improving and maintaining physical health.” That is, the second question is based on the answer to the first question and continues with the “why” or “how” question, and the third question is based on the answer to the second question and so on. There were some participants who entered into a more abstract way of thinking after the four consecutive “why” questions; this was the high-construal group. Other participants entered into a more concrete and specific way of thinking after four consecutive “how” questions; this was the low-construal group. Then, the participants completed the Behavior Identification Form for the manipulation test [80]. Specifically, the participants were asked to choose one of two possible options (one represents a high construal level, and the other represents a low construal level) for 10 behaviors [81]. We coded an answer as 0 (1) if the participant chose the low (high) construal level option (see Supplementary Material D). A higher score means a higher construal level. We found that the participants who answered “why” had a higher construal level, and those who answered “how” had a lower construal level (M _low_ = 2.15, SD = 2.090; M _high_ = 3.03, SD = 2.365; t (59) = 2.241, *p* < 0.05).

Finally, a manipulation test of the functional descriptive text (*n* = 60) and emotional descriptive text (*n* = 60) was conducted. Similar to the descriptive text in Study 1, the functional descriptive text was “The product has passed ‘EU organic certification’. Contain at least 95% organic ingredients. Meet ‘Euro-leaf’ eco-label’s principles of natural production and ecological balance.” The emotional descriptive text was “Do you want a healthier, greener lifestyle? Then choose products that have passed ‘Euro-leaf’ organic certification! Let’s live in harmony with nature!” (see Supplementary Material C). The results showed that the manipulation of the descriptive text was successful (M _functional_ = 3.22, SD = 1.263; M _emotional_ = 5.17, SD = 1.137; t (59) = −7.268, *p* < 0.001).

#### 6.1.2. Procedure

Participants were asked to answer four consecutive “why” or “how” questions to prime them into the corresponding construal level. Then, the functional JPEI group viewed the “GRE” oatmeal packaging with the Euro-leaf eco-label and functional descriptive text; the emotional JPEI group viewed the “GRE” oatmeal packaging with the Euro-leaf eco-label and emotional descriptive text. Participants were then administered the same measures as in Studies 1 and 2 for cognitive fluency (α = 0.860), purchase intentions (α = 0.854), and demographic variables.

### 6.2. Results

#### 6.2.1. Cognitive Fluency

**Figure 5:**
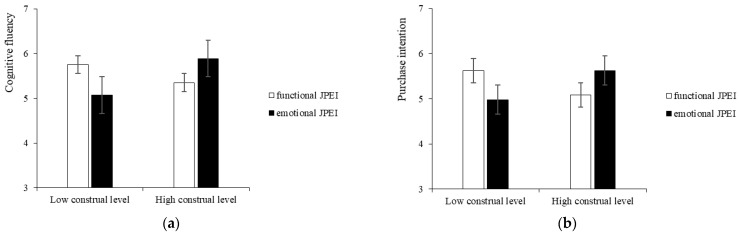
The interactive effect of the information type of JPEI and consumer’s construal level (Study 3). Note: (**a**) shows the interaction effect with cognitive fluency as the dependent variable; (**b**) shows the interaction effect with purchase intention as the dependent variable.

We conducted two-way ANOVA in SPSS 25.0 with cognitive fluency as the DV and information type and construal level as the IV. The interaction between information type and participants’ construal level in relation to cognitive fluency was significant (F (1, 310) = 25.481, *p* < 0.001, η_p_^2^ = 0.076). As shown in Figure 5a, for participants with a low construal level, functional JPEI had higher cognitive fluency than emotional JPEI (M _functional_ = 5.750, SD = 0.865; M _emotional_ = 5.071, SD = 1.099; F (1, 310) = 12.595, *p* < 0.001, η_p_^2^ = 0.039). For participants with a high construal level, cognitive fluency was higher for emotional JPEI than for functional JPEI (M _emotional_ = 5.890, SD = 0.988; M _functional_ = 5.352, SD = 1.107; F (1, 310) = 13.446, *p* < 0.001, η_p_^2^ = 0.042).

#### 6.2.2. Purchase Intention

We conducted two-way ANOVA in SPSS 25.0 with purchase intention as the DV and information type and construal level as the IV. The interaction effect between information type and participants’ construal level on purchase intentions was significant (F (1, 310) = 31.535, *p* < 0.001, η_p_^2^ = 0.092). As shown in Figure 5b, for participants with a low construal level, functional JPEI showed a higher purchase intentions than emotional JPEI (M _functional_ = 5.622, SD = 0.813; M _emotional_ = 4.976, SD = 0.938; F (1, 310) = 14.903, *p* < 0.001, η_p_^2^ = 0.046). For participants with a high construal level, emotional JPEI showed a higher purchase intentions than functional JPEI (M _emotional_ = 5.623, SD = 0.934; M _functional_ = 5.084, SD = 0.899; F (1, 310) = 17.586, *p* < 0.001, η_p_^2^ = 0.054).

#### 6.2.3. Moderated Mediation

**Figure 6:**
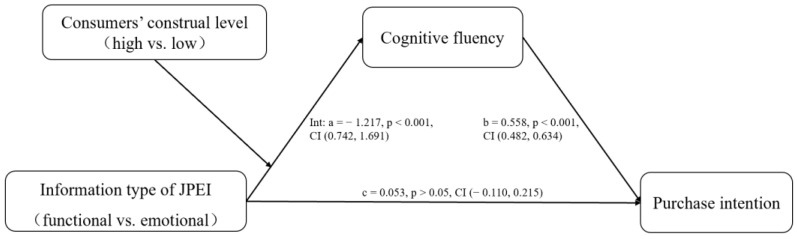
Results for the moderated mediation effect (Study 3).

We used PROCESS 3.3 in SPSS 25.0 to analyze the mediating role of cognitive fluency [75]. Bootstrapping analysis (samples = 5000, 95% CI, Model 7) showed that the mediating effect of cognitive fluency was significant (indirect effect = 0.679, SE = 0.141, 95% CI = 0.408 to 0.963, excluding 0). Specifically, in the low-construal group, information type had a significant effect on purchase intentions through cognitive fluency (indirect effect = −0.379, SE = 0.106, 95% CI = −0.590 to −0.174, excluding 0). In the high-construal group, information type also had a significant effect on purchase intentions through cognitive fluency (indirect effect = 0.300, SE = 0.088, 95% CI = 0.132 to 0.480, excluding 0). These results support H3a and H3b (see Figure 6).

### 6.3. Discussion

Study 3 demonstrated that matching the JPEI information type and consumers’ construal level could improve their cognitive fluency in eco-labels and subsequently increase their purchase intentions. Specifically, participants with a low construal level had higher cognitive fluency and purchase intentions when faced with functional JPEI; participants with a high construal level had higher cognitive fluency and purchase intentions when faced with emotional JPEI. This study provides evidence for what types of information should be added to eco-labels for consumers with low eco-label knowledge. The results of all hypothesis testing in this study are shown in Table 1.

## 7. General Discussion

### 7.1. Theoretical Contribution

First, we propose a new form of eco-label information presentation, which enriches the empirical research in the field of eco-labels. This study divided eco-label information presentation into two categories: SPEI and JPEI. This fills the gap in the research on eco-label information presentation or eco-label format while also enriching research on DCT. Some consumers have difficulty processing and understanding eco-labels [82]. However, there is insufficient research on how to solve this problem. We introduced DCT into the eco-label field and proposed adding descriptive text to eco-labels based on the cognitive perspective. Participants’ understanding of eco-labels was improved through the dual-coding JPEI approach. Previous research has similarly found that consumers usually prefer specific, detailed eco-information [83,84]. JPEI can also give consumers specific, detailed information, which reduces cognitive difficulty of eco-labels, especially for those who lack eco-label knowledge.

Second, we identified a spatial contiguity effect of JPEI, which not only provides a theoretical basis for the spatial distribution of eco-label information presentation, but also expands the application field of the spatial contiguity effect. In the past, the spatial contiguity effect has been mainly applied to multimedia learning [52,56], psychology [85], and other fields. We applied this theory to eco-labels to demonstrate the optimal spatial distance in JPEI. Previous research on eco-label positioning focused on the position of eco-labels on packaging based on the visibility perspective. For example, Gutierrez et al. [86] found that placing an eco-label in the center of a package better attracted visual attention. Carrero et al. [28], meanwhile, suggested that placing an eco-label in front of or near the nutritional information could make eco-labels more noticeable. Those studies, however, were mainly based on SPEI (i.e., a single eco-label icon), and they addressed visual attention to the eco-label but not the cognition of the eco-label. They also neglected the effect of the distance between the eco-label icon and other eco-label elements. We have, to some extent, filled this research gap. This study can also be extended to research on brand logos or other visual symbols. For example, based on our conclusions, for a brand logo with low popularity, it should be accompanied by a textual description of the brand, and the two should be placed close to each other.

Finally, we found a matching effect between the JPEI type and consumers’ construal level when consumers’ eco-label knowledge was low, which not only provides theoretical support for the design of eco-label information presentation content, but also extends the application of construal level theory. Previous research on eco-label information has compared the effectiveness of text and icons. For example, Rihn et al. [12] found that eco-label icons attract more visual attention and a relatively higher payment premium than textual eco-labels. However, no previous studies have explored the combined effect of icons and text or the explanatory role of eco-label text. Aiming to fill this gap, we classified descriptive eco-label text into functional and emotional categories and matched them with consumers’ construal levels to explore the effects on cognition and consumption behavior.

### 7.2. Managerial Implications

First, eco-label certification institutions should add descriptive text to help inform consumers and reduce their search costs. When a new eco-label is introduced to a market or region, consumers have less knowledge about it and have more difficulty recognizing and understanding it if the format is simple and information is limited. JPEI can not only increase the cognition of eco-labels but also educate consumers, thus promoting green consumption. Eco-label certification institutions should ensure the spatial contiguity of the eco-label and text when designing eco-labels. Additionally, eco-label certification institutions should use different types of information—namely, functional and emotional—to match different construal levels among consumers.

Second, governments should call on certification institutions to optimize eco-label design and convey more eco-label information to consumers. We showed that increasing consumers’ cognitive fluency in eco-labels can positively affect purchase intentions. Thus, popularizing eco-label knowledge and simplifying eco-label cognition for consumers can promote green consumption.

Finally, companies can choose eco-labels with more descriptive text, or they can independently add text adjacent to eco-labels when designing packaging to reduce consumers’ cognitive difficulties. Companies should also consider consumers’ construal level when establishing descriptive eco-label text. For consumers with a low construal level, functional text should be added; for those with a high construal level, emotional text should be used. Construal level can be either a long-term mindset or an induced temporary mindset [63]. Companies can use machine learning to quickly and effectively identify consumers with different construal levels based on their online behavior data [87]. Companies can also collect information from consumers and build consumer profiles to help identify consumers with different construal levels [60,88]. Alternatively, some elements can be used to induce consumers to form a specific construal level, such as using language or imagery that indicates the present or the future [63] or manipulating the visual height of advertising images [89].

### 7.3. Limitations and Future Research

This study has some limitations. First, we use a subjective measure for eco-label knowledge, and it is difficult to know whether the text information improved actual comprehension of the eco-label information. Future studies could use an objective measure of consumer eco-label knowledge to verify if the same results can be obtained. Second, our study was conducted in China, so it is uncertain whether the results can be generalized to other countries. Future studies could revalidate our results in other countries. Third, we mainly focused on eco-labels with low cognition, which may be in the early stages of development or newly introduced in a certain country, region, or market. We therefore need to further explore whether adding descriptive eco-label text will increase consumers’ cognitive load and cause some negative effects when eco-labels have reached the mature stage of development. Fourth, the descriptive eco-label text used in this study mainly included information such as the full name of the eco-label, certification institutions, certification standards, or principles. We need to explore whether too much descriptive text increases cognitive load or whether too little fails to improve cognitive fluency. In addition, given the limited space on packaging, would a QR code associated with the eco-label be more practical? Or would it not produce equivalent effects for low-knowledge consumers? Fifth, the complexity of other elements on the package can also affect how consumers respond to JPEI. For example, too many other information elements may interfere with the consumers’ processing of JPEI. Finally, some demographic variables may influence consumers’ willingness to purchase green products, such as gender, age, and education. This study did not take them into account as covariates in our model. Future studies could add this aspect to be considered.

## 8. Conclusions

This study’s findings can provide insights for improving the use of eco-labels. From the producer’s perspective, eco-labels are a tool for communicating the green attributes of products and services. However, from the consumer’s perspective, many eco-labels do not communicate effectively. We therefore proposed JPEI, which can improve consumers’ cognitive fluency in eco-labels. Study 1 confirmed H1a and H1b, that is, for consumers with low eco-label knowledge, JPEI enhanced cognitive fluency more so than SPEI. Study 2 further verified the effect of spatial distance (H2a and H2b). For consumers with low eco-label knowledge, spatially contiguous JPEI could improve cognitive fluency more than spatially partitioned JPEI. This in turn significantly affected purchase intentions, but there was no significant difference for consumers with high eco-label knowledge. Study 3 explored the effect of the JPEI type among consumers with low eco-label knowledge (H3a and H3b). Functional JPEI matched more with low-construal consumers, and emotional JPEI matched more with high-construal consumers. Such matching improved participants’ cognitive fluency in eco-labels and increased their willingness to purchase the product.

## Figures and Tables

**Table 1:** Summary of the findings.

**Hypotheses**	**Study**	Results
Eco-label information presentation (SPEI vs. JPEI) * consumers’ eco-label knowledge → Cognitive fluency		
H1a: Significant difference for low eco-label knowledge consumers	Study 1	√
H1b: No difference for high eco-label knowledge consumers	Study 1	√
Spatial distance of JPEI (spatially contiguous vs. spatially partitioned) * consumers’ eco-label knowledge → Cognitive fluency → Purchase intentions		
H2a: Significant difference for low eco-label knowledge consumers	Study 2	√
H2b: No difference for high eco-label knowledge consumers	Study 2	√
Information type of JPEI (functional vs. emotional) * consumers’ construal level (high vs. low) → Cognitive fluency → Purchase intentions		
H3a: Functional JPEI for low-construal consumers	Study 3	√
H3b: Emotional JPEI for high-construal consumers	Study 3	√

Note: * means interactive effect.

## Data Availability

Due to the confidentiality of the subjects’ privacy, data can be obtained by contacting the authors.

## Supplementary Materials

The following supporting information can be downloaded at: www.mdpi.com/article/10.3390/ijerph192013713/s1. Supplementary Material A: The Stimuli of Study 1 and Follow-up Study; Supplementary Material B: The Stimuli of Study 2 and Follow-up studies; Supplementary Material C: The Stimuli of Study 3; Supplementary Material D: Measurement Items; Supplementary Material E: Demographic Variables; Figure S1: The interactive effect of eco-label information presentation and consumer’s eco-label knowledge (the follow-up of Study 1); Figure S2: The interactive effect of JPEI spatial distance and consumer’s eco-label knowledge on cognitive fluency (Study 2 Follow-up A); Figure S3: The interactive effect of JPEI spatial distance and consumer’s eco-label knowledge on purchase intention (Study 2 Follow-up A); Figure S4: Results of moderated mediation effect (Study 2 Follow-up A); Figure S5: The interactive effect of JPEI spatial distance and consumer’s eco-label knowledge on cognitive fluency (Study 2 Follow-up B); Figure S6: The interactive effect of JPEI spatial distance and consumer’s eco-label knowledge on purchase intention (Study 2 Follow-up B); Figure S7: Results of moderated mediation effect (Study 2 Follow-up B); Figure S8: The interactive effect of JPEI spatial distance and consumer’s eco-label knowledge on cognitive fluency (Study 2 Follow-up C); Figure S9: The interactive effect of JPEI spatial distance and consumer’s eco-label knowledge on purchase intention (Study 2 Follow-up C); Figure S10: Results of moderated mediation effect (Study 2 Follow-up C); Table S1. Demographic Variables.

## References

[1] Donato C., Adıgüzel F. (2022). Visual Complexity of Eco-Labels and Product Evaluations in Online Setting: Is Simple Always Better?. J. Retail. Consum. Serv..

[2] Horne R.E. (2009). Limits to Labels: The Role of Eco-Labels in the Assessment of Product Sustainability and Routes to Sustainable Consumption. Int. J. Consum. Stud..

[3] Sigurdsson V., Larsen N.M., Pálsdóttir R.G., Folwarczny M., Menon R.G.V., Fagerstrøm A. (2022). Increasing the Effectiveness of Ecological Food Signaling: Comparing Sustainability Tags with Eco-Labels. J. Bus. Res..

[4] Taufique K.M.R., Polonsky M.J., Vocino A., Siwar C. (2019). Measuring Consumer Understanding and Perception of Eco-Labelling: Item Selection and Scale Validation. Int. J. Consum. Stud..

[5] Song L., Lim Y., Chang P., Guo Y., Zhang M., Wang X., Yu X., Lehto M.R., Cai H. (2019). Ecolabel’s Role in Informing Sustainable Consumption: A Naturalistic Decision Making Study Using Eye Tracking Glasses. J. Clean. Prod..

[6] Bratt C., Hallstedt S., Robèrt K.-H., Broman G., Oldmark J. (2011). Assessment of Eco-Labelling Criteria Development from a Strategic Sustainability Perspective. J. Clean. Prod..

[7] Liu Q., Yan Z., Zhou J. (2017). Consumer Choices and Motives for Eco-Labeled Products in China: An Empirical Analysis Based on the Choice Experiment. Sustainability.

[8] Higgins K., Hutchinson W.G., Longo A. (2020). Willingness-to-Pay for Eco-Labelled Forest Products in Northern Ireland: An Experimental Auction Approach. J. Behav. Exp. Econ..

[9] Sörqvist P., Marsh J.E., Holmgren M., Hulme R., Haga A., Seager P.B. (2016). Effects of Labeling a Product Eco-Friendly and Genetically Modified: A Cross-Cultural Comparison for Estimates of Taste, Willingness to Pay and Health Consequences. Food Qual. Prefer..

[10] Waris I., Ahmed W. (2020). Empirical Evaluation of the Antecedents of Energy-Efficient Home Appliances: Application of Extended Theory of Planned Behavior. MEQ.

[11] Zhang X., Yin H., Zhao R. (2021). Consumer Willingness to Pay for Eco-Labels in China: A Choice Experiment Approach. J. Manag. Anal..

[12] Rihn A., Wei X., Khachatryan H. (2019). Text vs. Logo: Does Eco-Label Format Influence Consumers’ Visual Attention and Willingness-to-Pay for Fruit Plants? An Experimental Auction Approach. J. Behav. Exp. Econ..

[13] Taufique K.M.R., Siwar C., Talib B., Sarah F.H., Chamhuri N. (2014). Synthesis of Constructs for Modeling Consumers’ Understanding and Perception of Eco-Labels. Sustainability.

[14] Brécard D. (2014). Consumer Confusion over the Profusion of Eco-Labels: Lessons from a Double Differentiation Model. Resour. Energy Econ..

[15] Nugraha W.S., Chen D., Yang S.-H. (2022). The Effect of a Halal Label and Label Size on Purchasing Intent for Non-Muslim Consumers. J. Retail. Consum. Serv..

[16] Taufique K.M.R., Vocino A., Polonsky M.J. (2017). The Influence of Eco-Label Knowledge and Trust on pro-Environmental Consumer Behaviour in an Emerging Market. J. Strateg. Mark..

[17] Delmas M.A., Lessem N. (2015). Eco-Premium or Eco-Penalty? Eco-Labels and Quality in the Organic Wine Market. Bus. Soc..

[18] Donato C., D’Aniello A. (2021). Tell Me More and Make Me Feel Proud: The Role of Eco-Labels and Informational Cues on Consumers’ Food Perceptions. Brit. Food J..

[19] Dhir A., Sadiq M., Talwar S., Sakashita M., Kaur P. (2021). Why Do Retail Consumers Buy Green Apparel? A Knowledge-Attitude-Behaviour-Context Perspective. J. Retail. Consum. Serv..

[20] Kumar S., Murphy M., Talwar S., Kaur P., Dhir A. (2021). What Drives Brand Love and Purchase Intentions toward the Local Food Distribution System? A Study of Social Media-Based REKO (Fair Consumption) Groups. J. Retail. Consum. Serv..

[21] Paivio A. (1991). Dual Coding Theory: Retrospect And Current Status. Can. J. Psychol./Rev. Can. De Psychol..

[22] Matthes J., Wonneberger A., Schmuck D. (2014). Consumers’ Green Involvement and the Persuasive Effects of Emotional versus Functional Ads. J. Bus. Res..

[23] Vekiri I. (2002). What Is the Value of Graphical Displays in Learning?. Educ. Psychol. Rev..

[24] Mayer R.E., Gallini J.K. (1990). When Is an Illustration Worth Ten Thousand Words?. J. Educ. Psychol..

[25] Mayer R.E. (2017). Using Multimedia for E-learning. J. Comput. Assist. Learn..

[26] D’Souza C., Taghian M., Lamb P. (2006). An Empirical Study on the Influence of Environmental Labels on Consumers. Corp. Commun. Int. J..

[27] van Amstel M., Driessen P., Glasbergen P. (2008). Eco-Labeling and Information Asymmetry: A Comparison of Five Eco-Labels in the Netherlands. J. Clean. Prod..

[28] Carrero I., Valor C., Díaz E., Labajo V. (2021). Designed to Be Noticed: A Reconceptualization of Carbon Food Labels as Warning Labels. Sustainability.

[29] Sharma N.K., Kushwaha G.S. (2019). Eco-Labels: A Tool for Green Marketing or Just a Blind Mirror for Consumers. Electron. Green J..

[30] Thøgersen J. (2000). Psychological Determinants of Paying Attention to Eco-Labels in Purchase Decisions: Model Development and Multinational Validation. J. Consum. Policy.

[31] Schwarz N. (2004). Metacognitive Experiences in Consumer Judgment and Decision Making. J. Consum. Psychol..

[32] Lee A.Y., Labroo A.A. (2004). The Effect of Conceptual and Perceptual Fluency on Brand Evaluation. J. Mark. Res..

[33] Zou J., Tang Y., Qing P., Li H., Razzaq A. (2021). Donation or Discount: Effect of Promotion Mode on Green Consumption Behavior. Int. J. Environ. Res. Public Health.

[34] Jaud D.A., Melnyk V. (2020). The Effect of Text-Only versus Text-and-Image Wine Labels on Liking, Taste and Purchase Intentions. The Mediating Role of Affective Fluency. J. Retail. Consum. Serv..

[35] Kim S.-B., Kim K.J., Kim D.-Y. (2016). Exploring the Effective Restaurant CrM Ad: The Moderating Roles of Advertising Types and Social Causes. Int. J. Contemp. Hosp. Manag..

[36] Sahin S., Baloglu S., Topcuoglu E. (2020). The Influence of Green Message Types on Advertising Effectiveness for Luxury and Budget Hotel Segments. Cornell. Hosp. Q..

[37] Lee K., Choi J. (2019). Image-Text Inconsistency Effect on Product Evaluation in Online Retailing. J. Retail. Consum. Serv..

[38] Lee W.-K., Wu C.-J. (2018). Eye Movements in Integrating Geometric Text and Figure: Scanpaths and Given-New Effects. Int. J. Sci. Math. Educ..

[39] Mayer R.E., Sims V.K. (1994). For Whom Is a Picture Worth a Thousand Words? Extensions of a Dual-Coding Theory of Multimedia Learning. J. Educ. Psychol..

[40] Griffin M.M., Robinson D.H. (2000). Role of Mimeticism and Spatiality in Textual Recall. Contemp. Educ. Psychol..

[41] Schwartz N.H., Ellsworth L.S., Graham L., Knight B. (1998). Accessing Prior Knowledge to Remember Text: A Comparison of Advance Organizers and Maps. Contemp. Educ. Psychol..

[42] Dodd T.H., Laverie D.A., Wilcox J.F., Duhan D.F. (2005). Differential Effects of Experience, Subjective Knowledge, and Objective Knowledge on Sources of Information Used in Consumer Wine Purchasing. J. Hosp. Tour. Res..

[43] Wang H., Ma B., Bai R. (2019). How Does Green Product Knowledge Effectively Promote Green Purchase Intention?. Sustainability-.

[44] Chuang S.-C., Tsai C.-C., Cheng Y.-H., Sun Y.-C. (2009). The Effect of Terminologies on Attitudes Toward Advertisements and Brands: Consumer Product Knowledge as a Moderator. J. Bus. Psychol..

[45] Kumar P., Polonsky M., Dwivedi Y.K., Kar A. (2021). Green Information Quality and Green Brand Evaluation: The Moderating Effects of Eco-Label Credibility and Consumer Knowledge. Eur. J. Mark..

[46] Hong J., Sternthal B. (2010). The Effects of Consumer Prior Knowledge and Processing Strategies on Judgments. J. Mark. Res..

[47] Naderi I., Paswan A.K., Guzman F. (2018). Beyond the Shadow of a Doubt: The Effect of Consumer Knowledge on Restaurant Evaluation. J. Retail. Consum. Serv..

[48] Taufique K.M.R., Siwar C.B., Talib B.B.A., Chamhuri N. (2014). Modelling Consumers’ Environmental Responsibility and Understanding of Eco-Labels: A Conceptual Framework for Empirical Research in Malaysia. Int. J. Green Econ..

[49] Zhang Z., Qiao S., Chen Y., Zhang Z. (2022). Effects of Spatial Distance on Consumers’ Review Effort. Ann. Tour. Res..

[50] Florax M., Ploetzner R. (2010). What Contributes to the Split-Attention Effect? The Role of Text Segmentation, Picture Labelling, and Spatial Proximity. Learn. Instr..

[51] Ginns P. (2006). Integrating Information: A Meta-Analysis of the Spatial Contiguity and Temporal Contiguity Effects. Learn. Instr..

[52] Schroeder N.L., Cenkci A.T. (2018). Spatial Contiguity and Spatial Split-Attention Effects in Multimedia Learning Environments: A Meta-Analysis. Educ. Psychol. Rev..

[53] Glaser M., Knoos M., Schwan S. (2020). The Closer, the Better? Processing Relations between Picture Elements in Historical Paintings. J. Eye Mov. Res..

[54] Cierniak G., Scheiter K., Gerjets P. (2009). Explaining the Split-Attention Effect: Is the Reduction of Extraneous Cognitive Load Accompanied by an Increase in Germane Cognitive Load?. Comput. Hum. Behav..

[55] Hu J., Zhang J. (2021). The Effect of Cue Labeling in Multimedia Learning: Evidence From Eye Tracking. Front. Psychol..

[56] Mayer R.E. (2003). The Promise of Multimedia Learning: Using the Same Instructional Design Methods across Different Media. Learn. Instr..

[57] Pihko E., Virtanen A., Saarinen V.-M., Pannasch S., Hirvenkari L., Tossavainen T., Haapala A., Hari R. (2011). Experiencing Art: The Influence of Expertise and Painting Abstraction Level. Front. Hum. Neurosci..

[58] Trope Y., Liberman N. (2010). Construal-Level Theory of Psychological Distance. Psychol. Rev..

[59] Dhar R., Kim E.Y. (2007). Seeing the Forest or the Trees: Implications of Construal Level Theory for Consumer Choice. J. Consum. Psychol..

[60] Dogan M., Erdogan B.Z. (2020). Effects of Congruence between Individuals’ and Hotel Commercials’ Construal Levels on Purchase Intentions. J. Hosp. Market. Manag..

[61] Wright S., Manolis C., Brown D., Guo X., Dinsmore J., Chiu C.-Y.P., Kardes F.R. (2012). Construal-Level Mind-Sets and the Perceived Validity of Marketing Claims. Market. Lett..

[62] Kim K., Lee S., Choi Y.K. (2019). Image Proximity in Advertising Appeals: Spatial Distance and Product Types. J. Bus. Res..

[63] Gong S., Sheng G., Peverelli P., Dai J. (2021). Green Branding Effects on Consumer Response: Examining a Brand Stereotype-Based Mechanism. J. Prod. Brand Manag..

[64] Hartmann P., Apaolaza Ibáñez V., Forcada Sainz F.J. (2005). Green Branding Effects on Attitude: Functional versus Emotional Positioning Strategies. Mark. Intell. Plan..

[65] Searles K. (2010). Feeling Good and Doing Good for the Environment: The Use of Emotional Appeals in pro-Environmental Public Service Announcements. Appl. Environ. Educ. Commun..

[66] Gong S., Lu J.G., Schaubroeck J.M., Li Q., Zhou Q., Qian X. (2020). Polluted Psyche: Is the Effect of Air Pollution on Unethical Behavior More Physiological or Psychological?. Psychol. Sci..

[67] Gai P.J., Puntoni S. (2021). Language and Consumer Dishonesty: A Self-Diagnosticity Theory. J. Consum. Res..

[68] Zhou Y., Fei Z., He Y., Yang Z. (2022). How Human–Chatbot Interaction Impairs Charitable Giving: The Role of Moral Judgment. J. Bus. Ethics.

[69] Tan Q., Imamura K., Nagasaka K., Inoue M. (2019). Effects of Eco-Label Knowledge on Chinese Consumer Preferences for Certified Wood Flooring: A Case Study in Chongqing City. Forest. Prod. J..

[70] Kwon O., Kim C., Kim G. (2013). Factors Affecting the Intensity of Emotional Expressions in Mobile Communications. Online Inform. Rev..

[71] McAndrew F.T., De Jonge C.R. (2011). Electronic Person Perception: What Do We Infer About People from the Style of Their E-Mail Messages?. Soc. Psychol. Pers. Sci..

[72] Aagerup U., Frank A.-S., Hultqvist E. (2019). The Persuasive Effects of Emotional Green Packaging Claims. Brit Food J..

[73] Chang C. (2004). The Interplay of Product Class Knowledge and Trial Experience in Attitude Formation. J. Advert..

[74] Lee A.Y., Aaker J.L. (2004). Bringing the Frame into Focus: The Influence of Regulatory Fit on Processing Fluency and Persuasion. J. Personal. Soc. Psychol..

[75] Hayes A.F. (2017). Introduction to Mediation, Moderation, and Conditional Process Analysis: A Regression-Based Approach.

[76] Spiller S.A., Fitzsimons G.J., Lynch J.G., Mcclelland G.H. (2013). Spotlights, Floodlights, and the Magic Number Zero: Simple Effects Tests in Moderated Regression. J. Mark. Res..

[77] Dodds W.B., Monroe K.B., Grewal D. (1991). Effects of Price, Brand, and Store Information on Buyers’ Product Evaluations. J. Mark. Res..

[78] Shimokawa S., Hu D., Li D., Cheng H. (2021). The Urban–Rural Gap in the Demand for Food Safety in China: The Role of Food Label Knowledge. Agric. Econ..

[79] Freitas A.L., Gollwitzer P., Trope Y. (2004). The Influence of Abstract and Concrete Mindsets on Anticipating and Guiding Others’ Self-Regulatory Efforts. J. Exp. Soc. Psychol..

[80] Vallacher R.R., Wegner D.M. (1989). Levels of Personal Agency: Individual Variation in Action Identification. J. Pers. Soc. Psychol..

[81] Septianto F., Lee M.S.W., Putra P.G. (2021). Everyday “Low Price” or Everyday “Value”? The Interactive Effects of Framing and Construal Level on Consumer Purchase Intentions. J. Retail. Consum. Serv..

[82] Cohen M.A., Vandenbergh M.P. (2012). The Potential Role of Carbon Labeling in a Green Economy. Energy Econ..

[83] Cho Y.-N. (2015). Different Shades of Green Consciousness: The Interplay of Sustainability Labeling and Environmental Impact on Product Evaluations. J. Bus. Ethics.

[84] Engels S.V., Hansmann R., Scholz R.W. (2010). Toward a Sustainability Label for Food Products: An Analysis of Experts’ and Consumers’ Acceptance. Ecol. Food Nutr..

[85] Roodenrys K., Agostinho S., Roodenrys S., Chandler P. (2012). Managing One’s Own Cognitive Load when Evidence of Split Attention Is Present. Appl. Cogn. Psych..

[86] Gutierrez A.M.J., Chiu A.S.F., Seva R. (2020). A Proposed Framework on the Affective Design of Eco-Product Labels. Sustainability.

[87] Wang X., Liu Y., Wang S., Chen H.A. (2022). Keep It Vague? New Product Preannouncement, Regulatory Focus, and Word-of-Mouth. J. Retail. Consum. Serv..

[88] Yao F.-S., Shao J.-B., Zhang H. (2021). Is Creative Description Always Effective in Purchase Intention? The Construal Level Theory as a Moderating Effect. Front. Psychol..

[89] Roose G., Vermeir I., Geuens M., Van Kerckhove A. (2019). A Match Made in Heaven or down under? The Effectiveness of Matching Visual and Verbal Horizons in Advertising. J. Consum. Psychol..

