# Supplementary material for: Telling You More Fluently: Effect of the Joint Presentation of Eco-Label Information on Consumers’ Purchase Intention
Source: Int J Environ Res Public Health. 2022 Oct 21;19(20):13713. doi: 10.3390/ijerph192013713 (PMC9603581; doi:10.3390/ijerph192013713)
Supplement: Supplementary file 1 [file ijerph-19-13713-s001.zip › ijerph-1971307-supplementary.pdf]

International Journal of
*Environmental Research
and Public Health*

MDPI

Supplemetary Materials

Telling You More Fluently: Effect of the Joint Presentation of Eco-Label Information on Consumers' Purchase Intention

Xingyuan Wang, Yingying Du *, Yun Liu and Shuyang Wang

School of Management, Shandong University, Jinan 250100, China
* Correspondence: yingdu@mail.sdu.edu.cn

Contents

Supplementary Material A. The Stimuli of Study 1 and Follow-up Study 2
Supplementary Material B. The Stimuli of Study 2 and Follow-up studies......................... 5
Study 2 Follow-up A.. 6
Study 2 Follow-up B 9
Study 2 Follow-up C.. 12
Supplementary Material C. The Stimuli of Study 3.. 15
Supplementary Material D. Measurement Items... 17
Supplementary Material E. Demographic Variables 19

Supplementary Material A. The Stimuli of Study 1 and Follow-up study

The Stimuli of Study 1

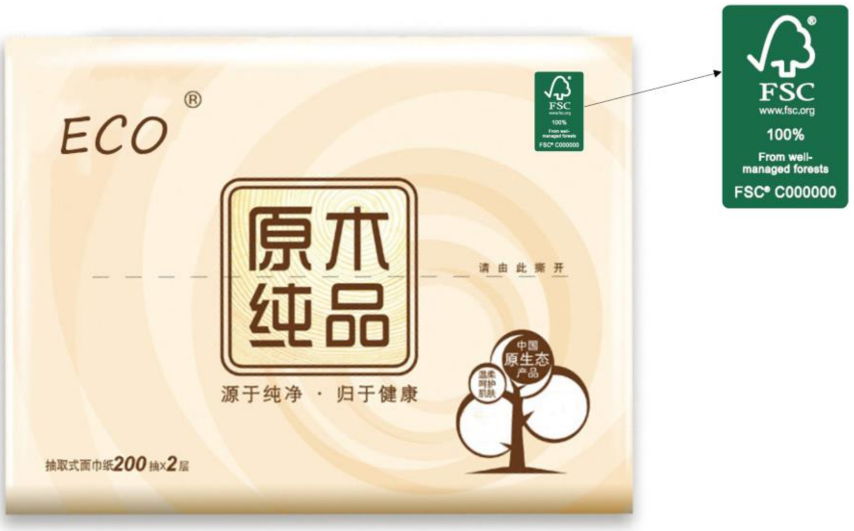

SPEI (Study 1)

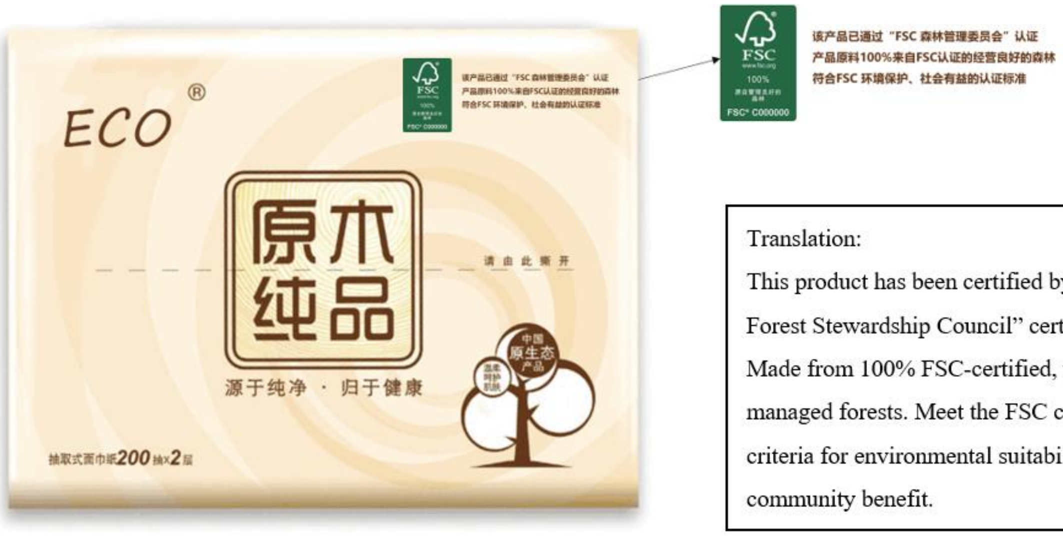

Translation:
This product has been certified by the “FSC Forest Stewardship Council” certification. Made from 100% FSC-certified, well-managed forests. Meet the FSC certification criteria for environmental suitability and community benefit.

JPEI (Study 1)

Follow-up Study

We recruited 234 participants for a study similar to Study 1 (see Supplementary Material E for demographic profiles of participants). The only difference was the descriptive text for the JPEI group. This follow-up study used the emotional descriptive text from the pre-test of Study 1. The stimuli were as follows.

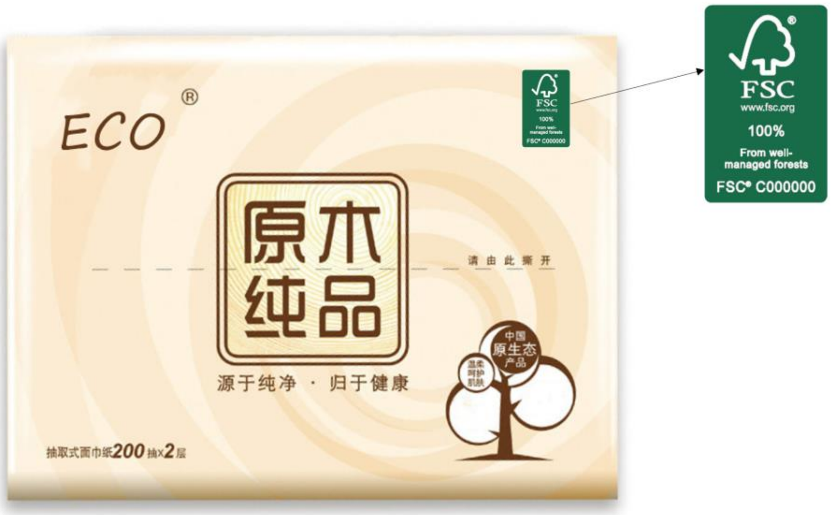

SPEI (Follow-up of Study 1)

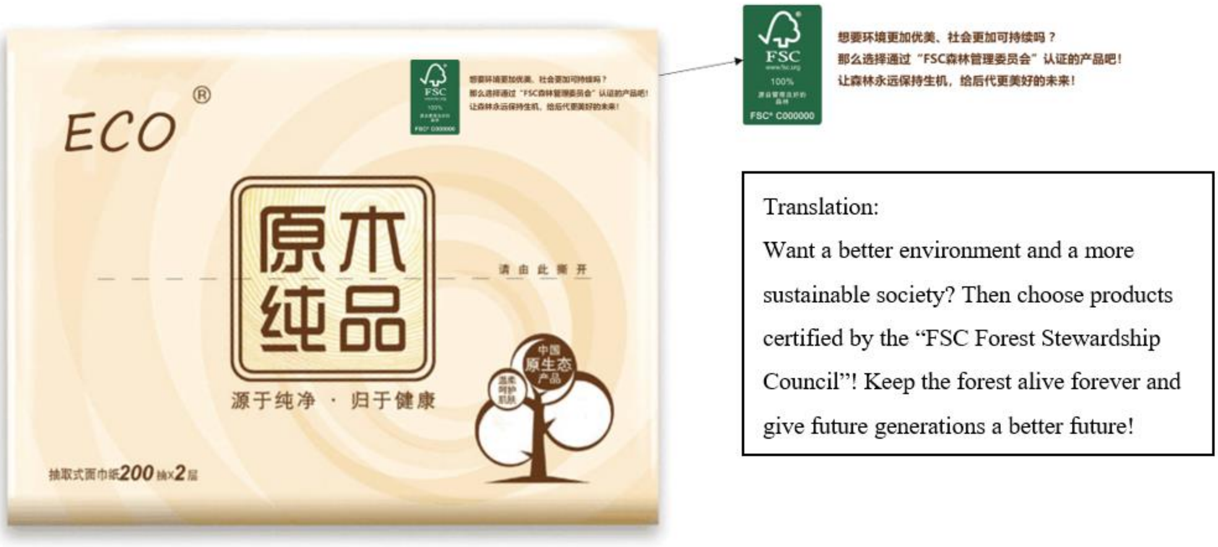

JPEI (Follow-up of Study 1)

Results

The results showed that the interaction between eco-label information presentation and consumers' eco-label knowledge on cognitive fluency is also significant (b = −0.235, SE = 0.076, t = −3.118, p < 0.01). The Johnson–Neyman test showed that eco-label information presentation had a significant positive effect on cognitive fluency when consumers' eco-label knowledge was equal to or lower than 3.998 (p = 0.001 to 0.05); when consumers' eco-label knowledge was higher than 3.998, the effect of eco-label information presentation on cognitive fluency was not significant (p = 0.05 to 0.891, $B_{JN = 3.998}$ = 0.224, SE = 0.114), supporting H1a and H1b (see Figure S1).

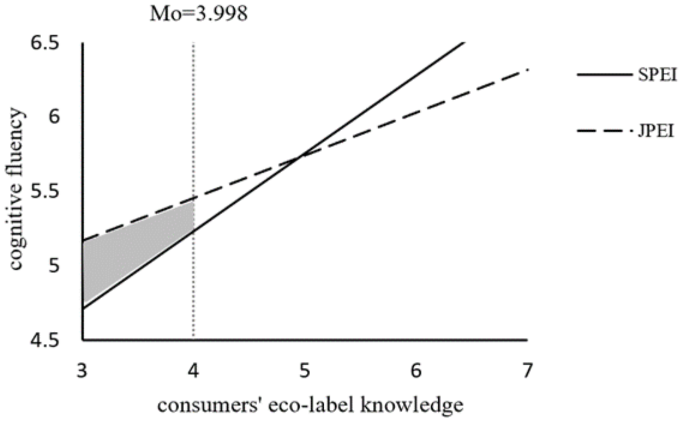

Figure S1. The interactive effect of eco-label information presentation and consumer's eco-label knowledge (the follow-up of Study 1). Note: the shaded area is the Johnson–Neyman significant area.

Supplementary Material B. The Stimuli of Study 2 and Follow-up Studies

The Stimuli of Study 2

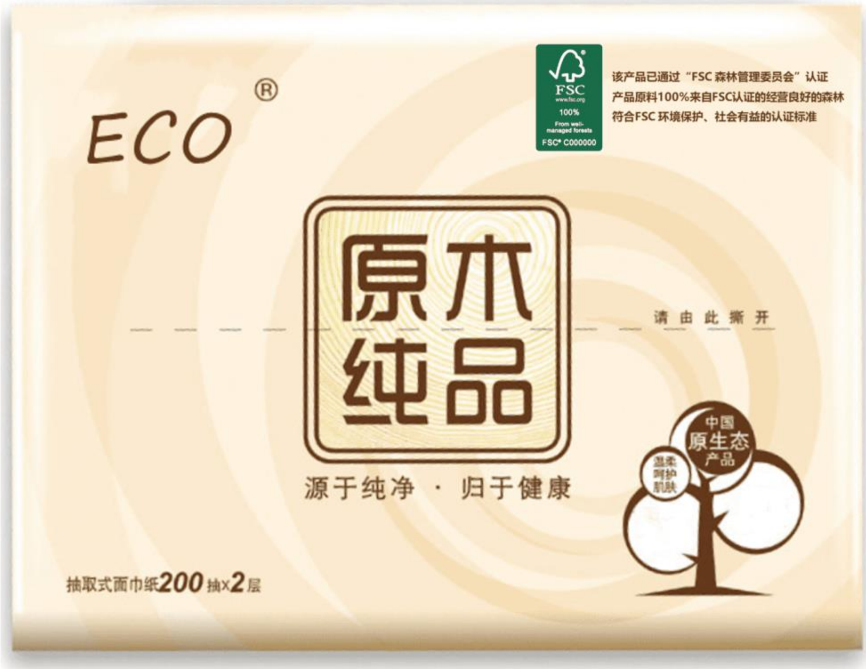

Spatially contiguous JPEI (Study 2)

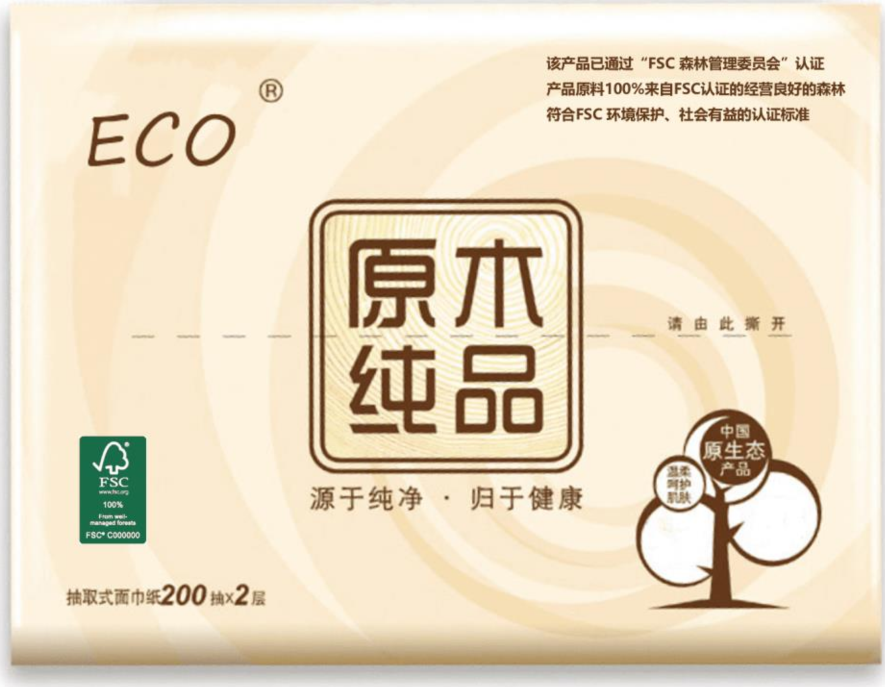

Spatially partitioned JPEI (Study 2)

Study 2 Follow-up A

We recruited 200 participants for a study similar to Study 2 (see Supplementary Material E for demographic profiles of participants). The only difference was the descriptive text. This follow-up study used the emotional descriptive text from the pre-test of Study 1. The stimuli were as follows.

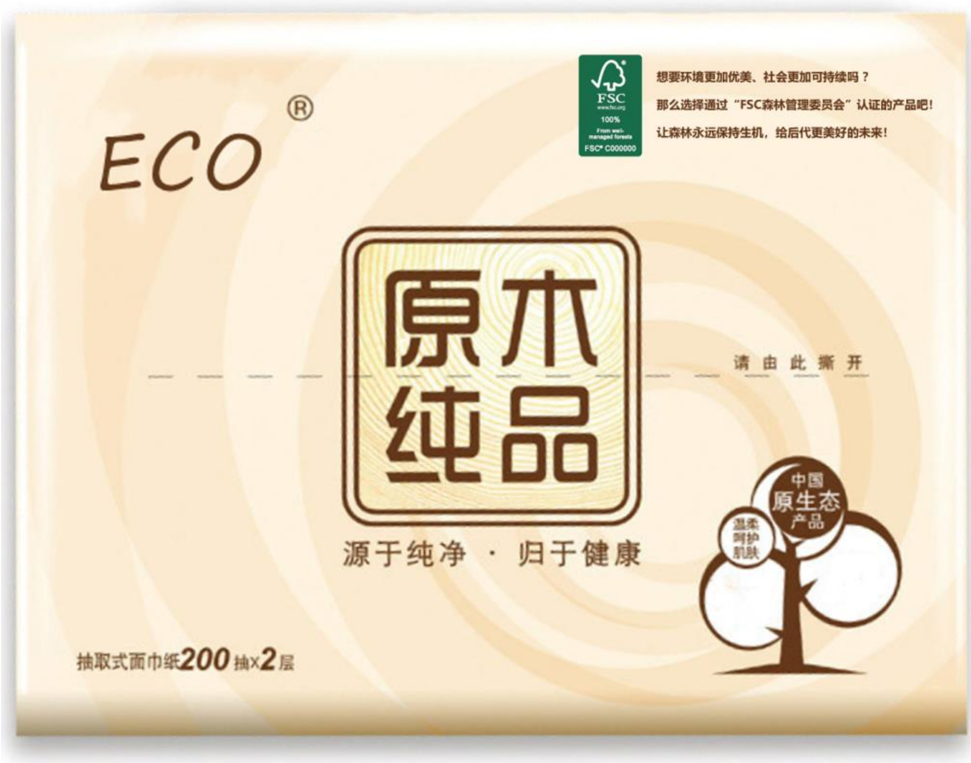

Spatially contiguous JPEI (Study 2 Follow-up A)

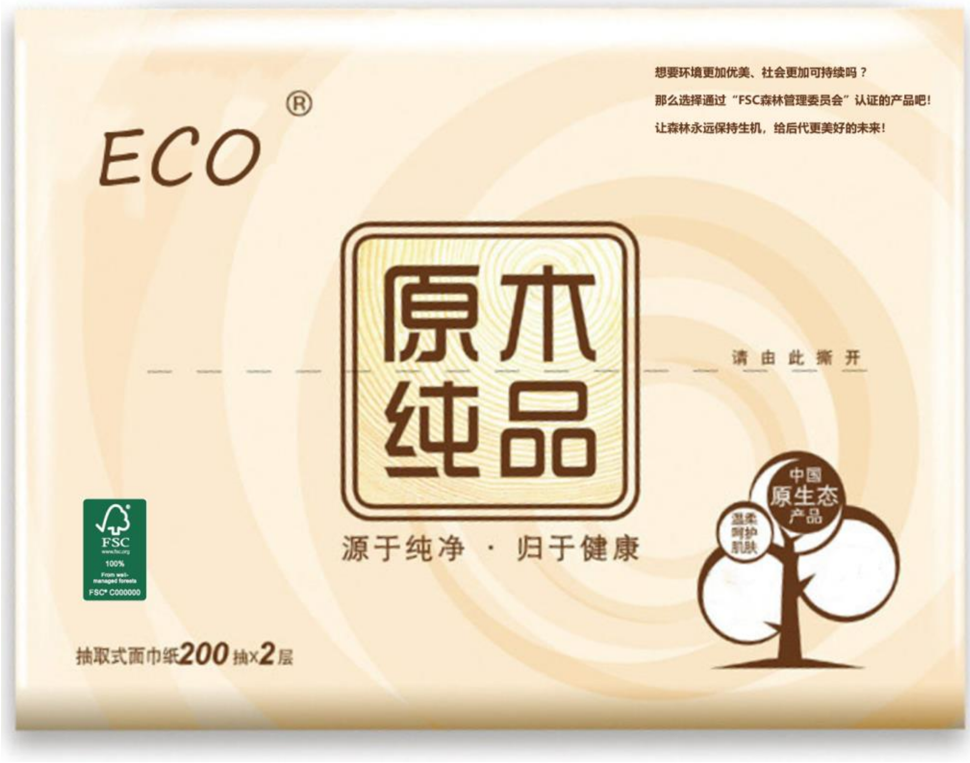

Spatially partitioned JPEI (Study 2 Follow-up A)

Manipulation Test

Participants perceived the spatially partitioned group as more distant than the spatially contiguous group ($M_{partitioned} = 5.142$, $SD = 1.670$; $M_{contiguous} = 3.309$, $SD = 1.201$; $t(198) = 8.811$, $p < 0.05$).

Cognitive Fluency

The interaction between spatial distance and consumers' eco-label knowledge on cognitive fluency was significant ($b = -0.253$, $SE = 0.075$, $t = -3.388$, $p < 0.001$). The Johnson–Neyman test showed that spatial distance had a significant positive effect on cognitive fluency when consumers' eco-label knowledge was equal to or lower than 4.4342 ($p = 0$ to 0.05); when consumers' eco-label knowledge was higher than 4.4342, the effect of spatial distance on cognitive fluency was not significant ($p = 0.05$ to 0.993, $B_{JN = 4.4342} = 0.224$, $SE = 0.113$) (see Figure S2).

Purchase Intention

The interaction between spatial distance and consumers' eco-label knowledge on purchase intention is also significant ($b = -0.121$, $SE = 0.054$, $t = -2.238$, $p < 0.05$). The Johnson–Neyman test showed that spatial distance had a significant positive effect on purchase intention when consumers' eco-label knowledge was equal to or lower than 5.0351 ($p = 0$ to 0.05); when consumers' eco-label knowledge was higher than 5.0351, the effect of spatial distance on purchase intention was not significant ($p = 0.05$ to 0.996, $B_{JN = 5.0531} = 0.172$, $SE = 0.087$) (see Figure S3).

Moderated mediation

PROCESS 3.3 (samples = 5000, 95% CI, Model 7) showed that the mediation of cognitive fluency was significant (indirect effect = −0.071, $SE = 0.028$, 95% $CI = -0.129$ to −0.019, excluding 0). In the low eco-label knowledge group, the spatial distance of JPEI had a significant effect on purchase intention through cognitive fluency (indirect effect = 0.170, $SE = 0.071$, 95% $CI = 0.041$ to 0.317, excluding 0). In the high eco-label knowledge group, the spatial distance of JPEI had a nonsignificant effect on purchase intention through cognitive fluency (indirect effect = −0.046, $SE = 0.032$, 95% $CI = -0.113$ to 0.012, including 0) (see Figure S4). The results support H2a and H2b.

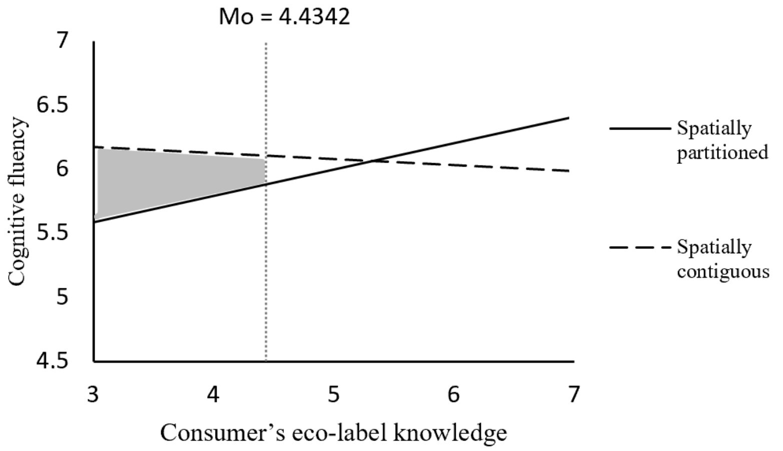

Figure S2. The interactive effect of JPEI spatial distance and consumer's eco-label knowledge on cognitive fluency (Study 2 Follow-up A). Note: the shaded area is the Johnson–Neyman significant area.

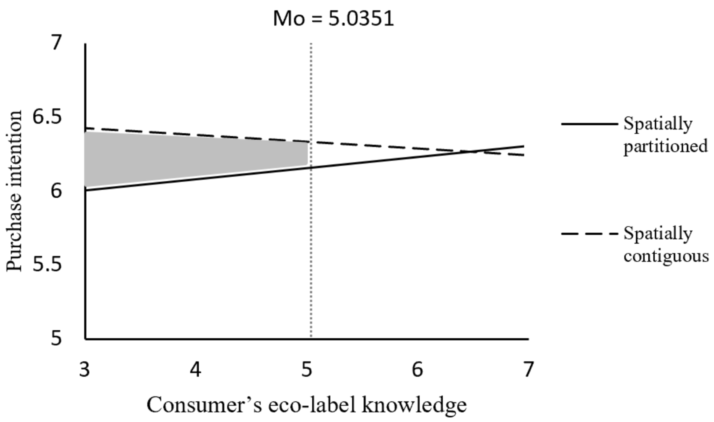

Figure S3. The interactive effect of JPEI spatial distance and consumer's eco-label knowledge on purchase intention (Study 2 Follow-up A). Note: the shaded area is the Johnson–Neyman significant area.

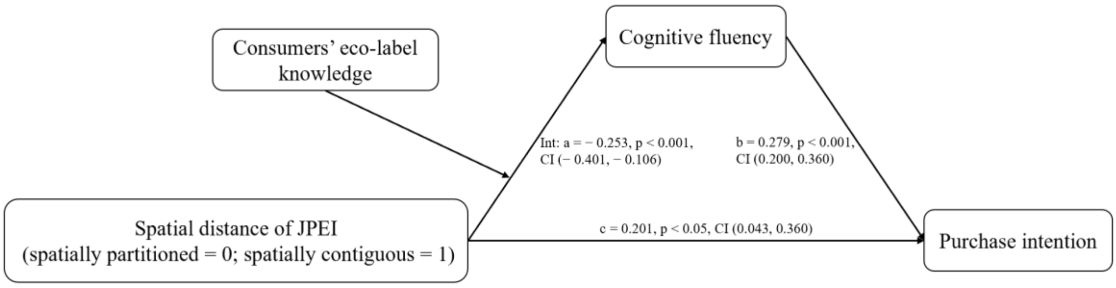

Figure S4. Results of moderated mediation effect (Study 2 Follow-up A).

Study 2 Follow-up B

The results we observe may be explained by the specific location of the information, rather than the spatial distance. Thus, we changed the relative position of the eco-label and the text and verified again. In this study, we placed both the eco-label and the descriptive text at the top of the package but separated them for the spatially partitioned JPEI group. We recruited 211 participants for a study similar to Study 2 (see Supplementary Material E for demographic profiles of participants). The stimuli were as follows.

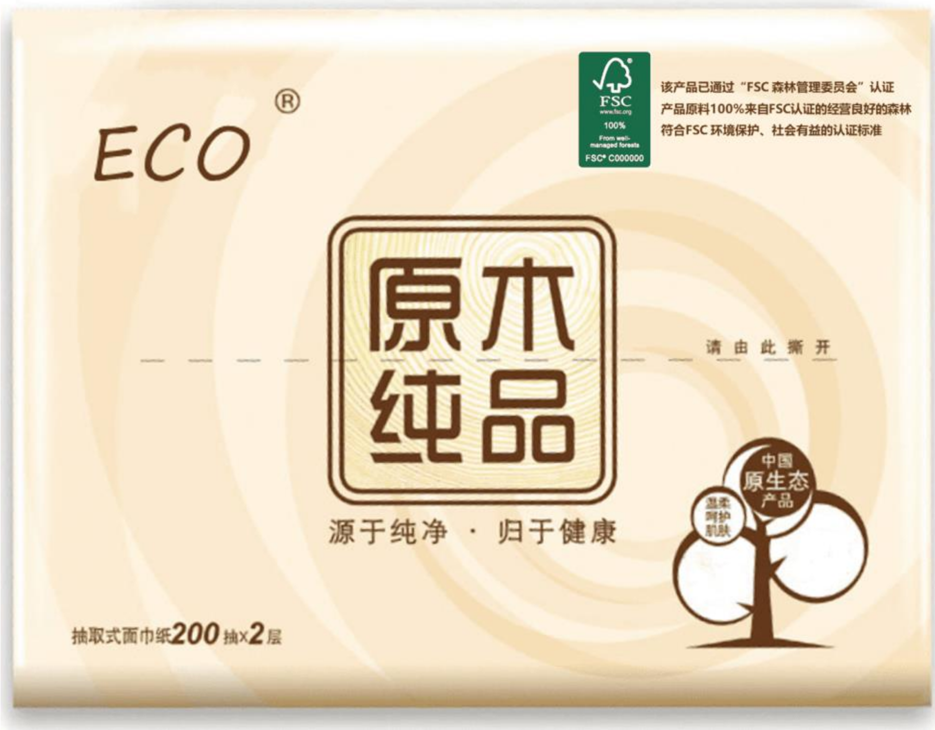

Spatially contiguous JPEI (Study 2 Follow-up B)

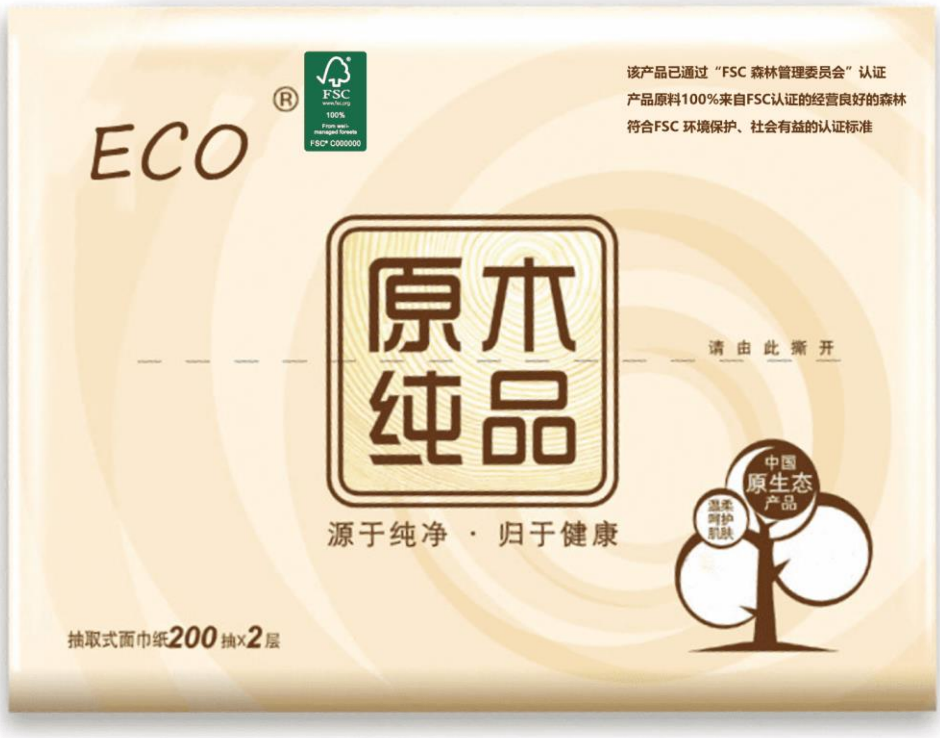

Spatially partitioned JPEI (Study 2 Follow-up B)

Manipulation Test

Participants perceived the spatially partitioned group as more distant than the spatially contiguous group ($M_{\text{partitioned}} = 5.260$, $SD = 1.013$; $M_{\text{contiguous}} = 3.100$, $SD = 1.235$; $t\ (209) = 13.948$, $p < 0.05$).

Cognitive Fluency

The interaction between spatial distance and consumers' eco-label knowledge on cognitive fluency was significant ($b = -0.216$, $SE = 0.080$, $t = -2.699$, $p < 0.01$). The Johnson–Neyman results showed that spatial distance had a significant positive effect on cognitive fluency when consumers' eco-label knowledge was equal to or lower than 4.8804 ($p = 0$ to 0.05); when consumers' eco-label knowledge was higher than 4.8804, the effect of spatial distance on cognitive fluency was not significant ($p = 0.05$ to 0.891, $B_{JN = 4.8804} = 0.254$, $SE = 0.129$) (see Figure S5).

Purchase Intention

The interaction between spatial distance and consumers' eco-label knowledge on purchase intention was also significant ($b = -0.129$, $SE = 0.063$, $t = -2.058$, $p < 0.05$). The Johnson–Neyman results showed that spatial distance had a significant positive effect on purchase intention when consumers' eco-label knowledge was equal to or lower than 4.4119 ($p = 0.004$ to 0.05); when consumers' eco-label knowledge was higher than 4.4119, the effect of spatial distance on purchase intention was not significant ($p = 0.05$ to 0.976, $B_{JN = 4.4119} = 0.186$, $SE = 0.094$) (see Figure S6).

Moderated Mediation

PROCESS 3.3 (samples = 5000, 95% CI, Model 7) showed that the mediation of cognitive fluency was significant (indirect effect = −0.060, $SE = 0.032$, 95% $CI = -0.130$ to −0.005, excluding 0). When consumers had low eco-label knowledge, the spatial distance of JPEI had a significant effect on purchase intention through cognitive fluency (indirect effect = 0.196, $SE = 0.084$, 95% $CI = 0.051$ to 0.387, excluding 0). When consumers had high eco-label knowledge, the spatial distance of JPEI had a nonsignificant effect on purchase intention through cognitive fluency (indirect effect = 0.014, $SE = 0.034$, 95% $CI = -0.052$ to 0.082, including 0) (see Figure S7). The results support H2a and H2b.

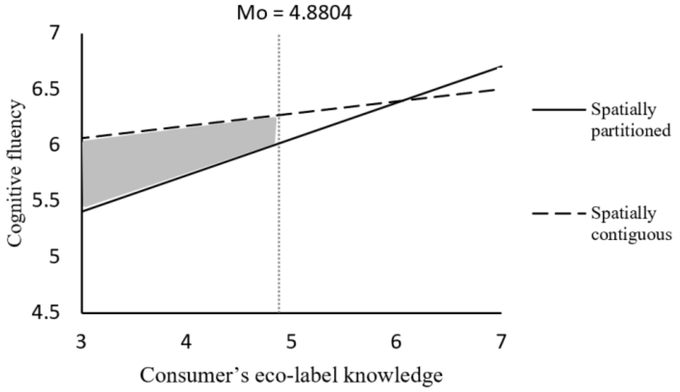

Figure S5. The interactive effect of JPEI spatial distance and consumer's eco-label knowledge on cognitive fluency (Study 2 Follow-up B). Note: the shaded area is the Johnson–Neyman significant area.

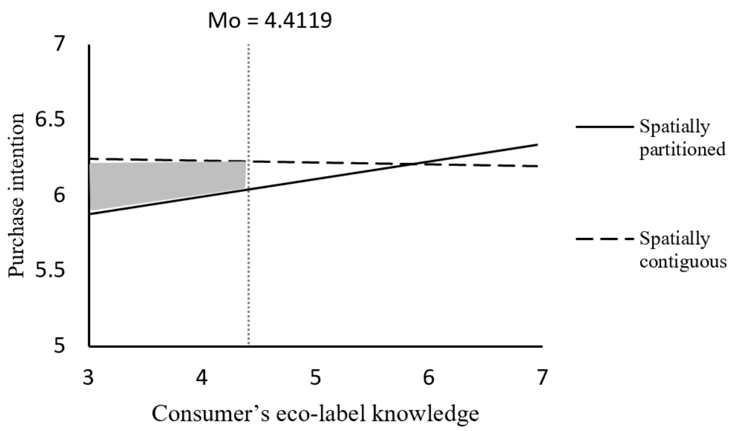

Figure S6. The interactive effect of JPEI spatial distance and consumer's eco-label knowledge on purchase intention (Study 2 Follow-up B). Note: the shaded area is the Johnson–Neyman significant area.

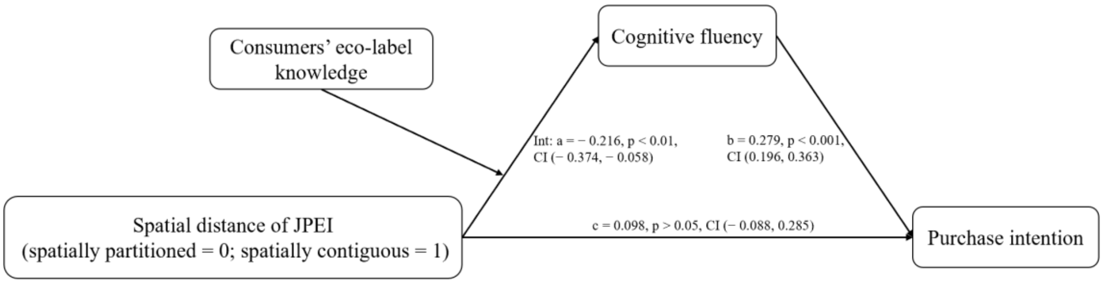

Figure S7. Results of moderated mediation effect (Study 2 Follow-up B).

Study 2 Follow-up C

In this study, we will change the position of the eco-label and text again to test our hypotheses. We place the eco-label in the top right corner of the package and the text in the bottom left corner of the package in the spatially partitioned JPEI group. We recruited 200 participants for a study similar to Study 2 (see Supplementary Material E for demographic profiles of participants). The stimuli were as follows.

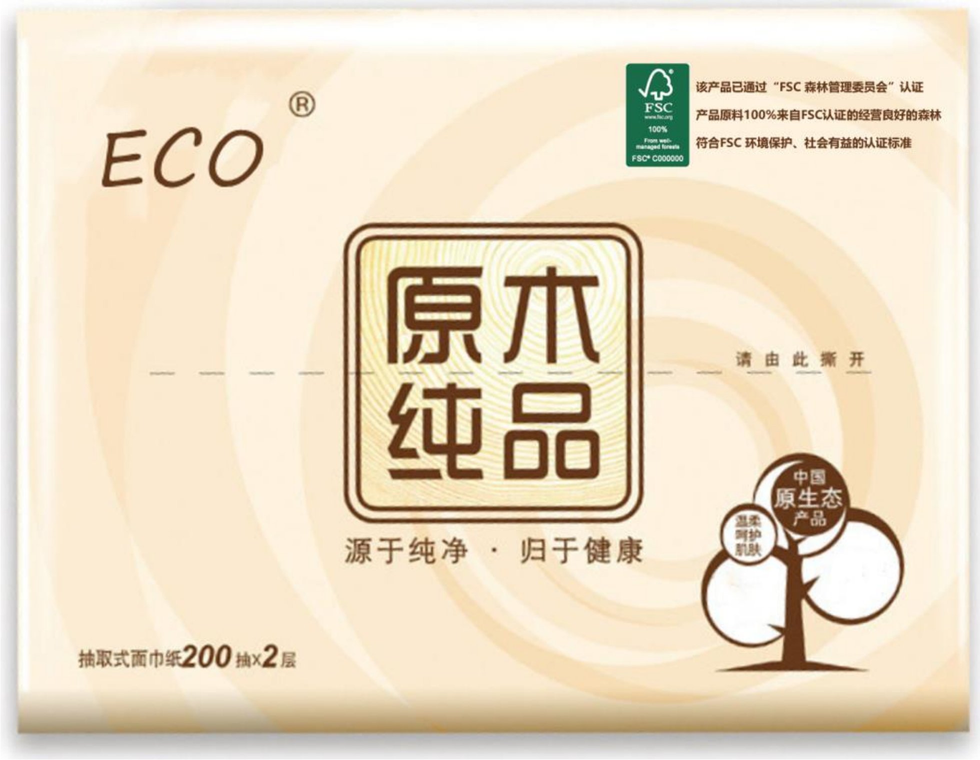

Spatially contiguous JPEI (Study 2 Follow-up C)

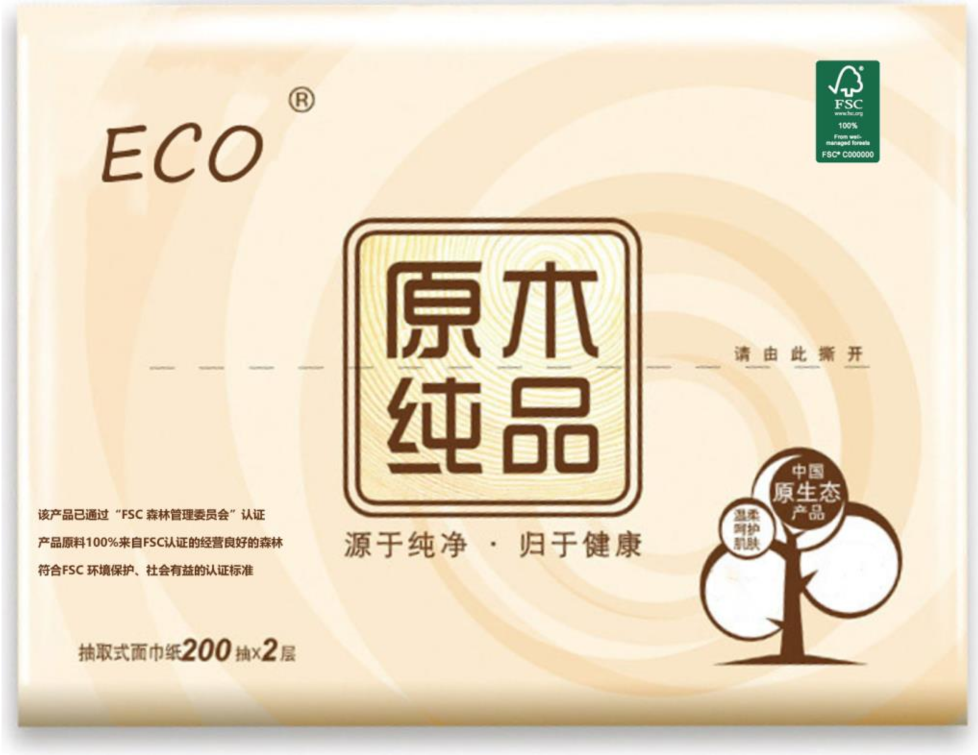

Spatially partitioned JPEI (Study 2 Follow-up C)

Manipulation Test

Participants perceived the spatially partitioned group as more distant than the spatially contiguous group ($M_{\text{partitioned}} = 4.340$, $SD = 1.919$; $M_{\text{contiguous}} = 3.510$, $SD = 1.251$; t (198) = 3.624, $p < 0.001$).

Cognitive Fluency

The interaction between spatial distance and consumers' eco-label knowledge on cognitive fluency was significant ($b = -0.234$, $SE = 0.090$, $t = -2.587$, $p < 0.05$). The Johnson–Neyman results showed that spatial distance had a significant positive effect on cognitive fluency when consumers' eco-label knowledge was equal to or lower than 5.4605 (p = 0 to 0.05); when consumers' eco-label knowledge was higher than 5.4605, the effect of spatial distance on cognitive fluency was not significant (p = 0.05 to 0.898, $B_{JN = 5.4605} = 0.334$, SE = 0.170) (see Figure S8).

Purchase Intention

The interaction between spatial distance and consumers' eco-label knowledge on purchase intention was also significant ($b = -0.154$, $SE = 0.076$, $t = -2.037$, $p < 0.05$). The Johnson–Neyman results showed that spatial distance had a significant positive effect on purchase intention when consumers' eco-label knowledge was equal to or lower than 5.0482 (p = 0 to 0.05); when consumers' eco-label knowledge was higher than 5.0482, the effect of spatial distance on purchase intention was not significant (p = 0.05 to 0.943, $B_{JN = 5.0482} = 0.246$, $SE = 0.125$) (see Figure S9).

Moderated Mediation

PROCESS 3.3 (samples = 5000, 95% CI, Model 7) showed that the mediation of cognitive fluency was significant (indirect effect = −0.093, SE = 0.048, 95% CI = −0.189 to −0.003, excluding 0). When consumers had low eco-label knowledge, the spatial distance of JPEI had a significant effect on purchase intention through cognitive fluency (indirect effect = 0.380, SE = 0.127, 95% CI = 0.158 to 0.659, excluding 0). When consumers had high eco-label knowledge, the spatial distance of JPEI had a nonsignificant effect on purchase intention through cognitive fluency (indirect effect =−0.103, SE = 0.080, 95% CI = −0.020 to 0.287, including 0) (see Figure S10). The results support H2a and H2b.

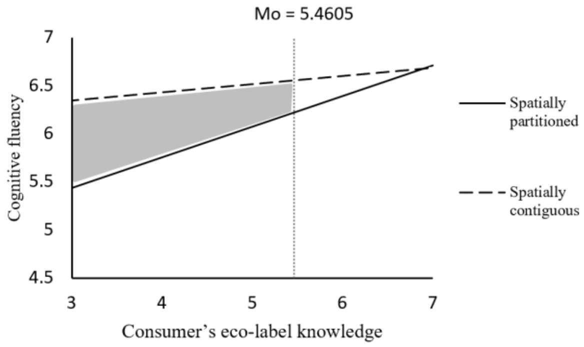

Figure S8. The interactive effect of JPEI spatial distance and consumer's eco-label knowledge on cognitive fluency (Study 2 Follow-up C). Note: the shaded area is the Johnson–Neyman significant area.

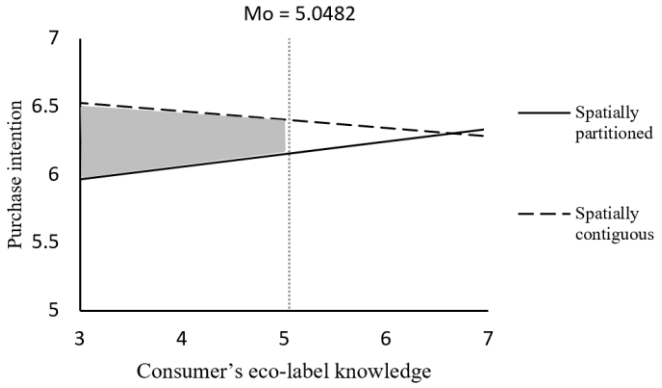

Figure S9. The interactive effect of JPEI spatial distance and consumer's eco-label knowledge on purchase intention (Study 2 Follow-up C). Note: the shaded area is the Johnson–Neyman significant area.

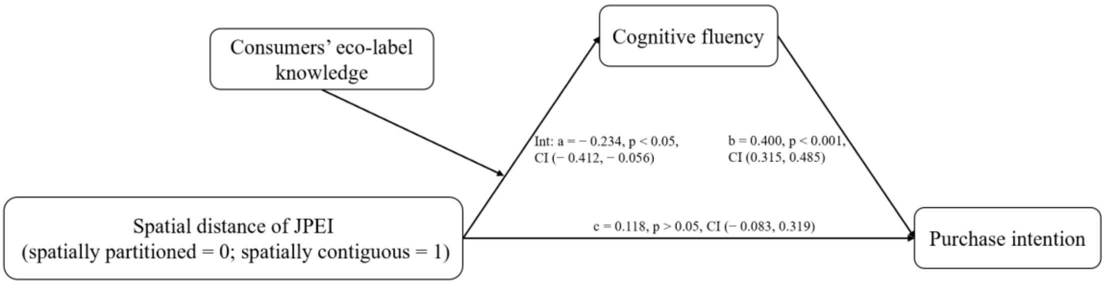

Figure S10. Results of moderated mediation effect (Study 2 Follow-up C).

Supplementary Material C. The Stimuli of Study 3

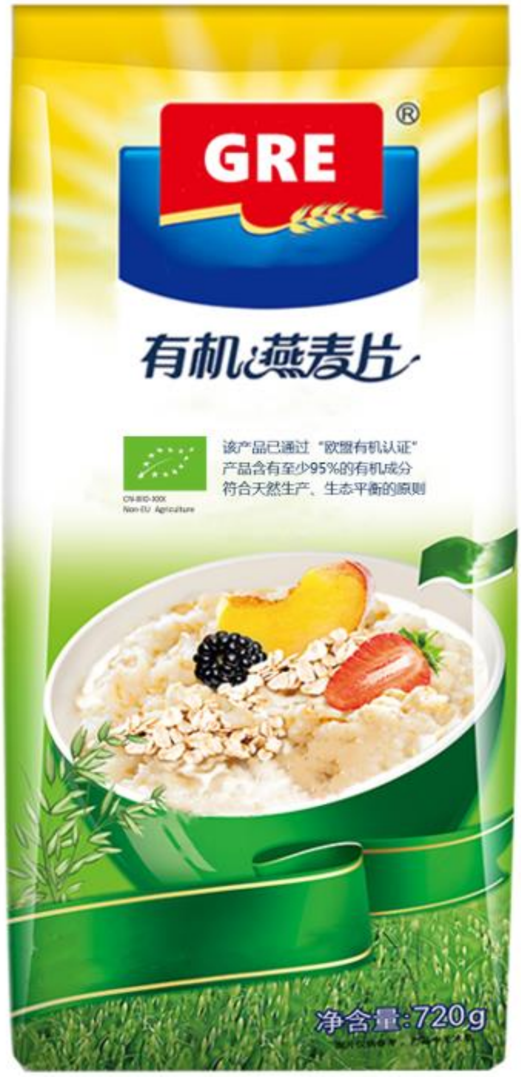

Translation:
The product has passed the "EU organic certification". Contain at least 95% organic ingredients. Meet "Euro-leaf" eco-label's principles of natural production and ecological balance.

Functional JPEI (Study 3)

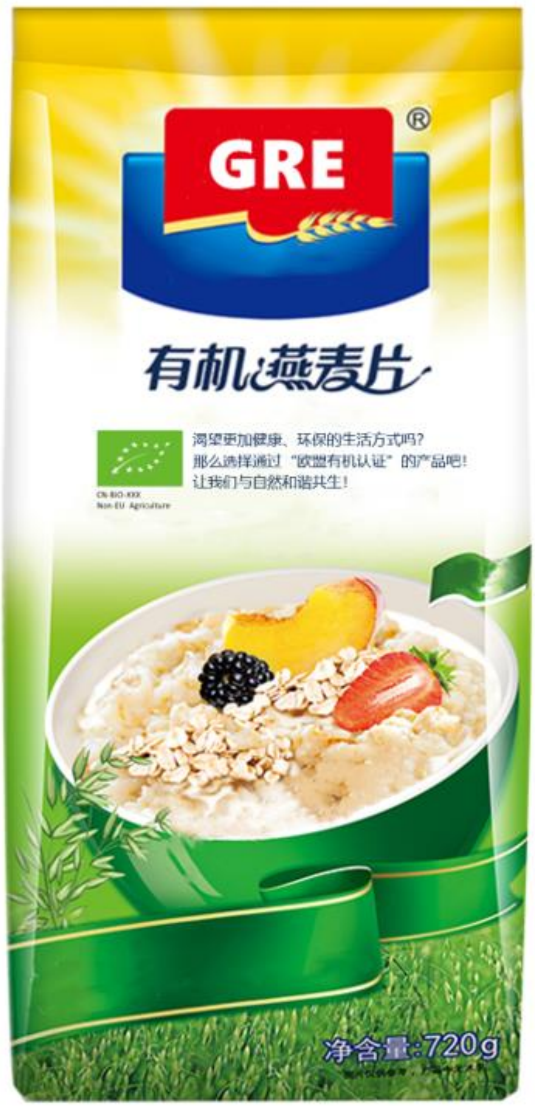

Translation:
Do you want a healthier, greener lifestyle? Then choose products that have passed the “Euro-leaf” organic certification! Let’ s live in harmony with nature!

Emotional JPEI (Study 3)

Supplementary Material D. Measurement Items

Consumers' eco-label knowledge: (Chang, 2004)

I think I know a lot about the eco-label.
I would consider myself an expert in terms of my knowledge of the eco-label.
I know more about the eco-label than my friends do.
I usually pay a lot of attention to the eco-label information on products.

Cognitive fluency: (Lee and Aaker, 2004)

I think the eco-label is easy to understand.
I think it is very simple to process the eco-label.
I can clearly understand the content of the eco-label.

Purchase intention: (Dodds et al., 1991)

I might consider buying the product.
My possibility of buying the product is high.
My willingness to buy the product is high.

Environmental concern: (Matthes et al., 2014)

I am concerned about the environment.
The condition of the environment affects the quality of my life.
I am willing to make sacrifices to protect the environment.
My actions impact the environment.

Construct level manipulation test: (Septianto et al., 2021)

Making a list

Getting organized (1)
Writing things down (0)

Reading

Gaining knowledge (1)
Following lines of print (0)

Joining the Army

Helping the Nation's defense (1)
Signing up (0)

Picking an apple

Getting something to eat (1)
Pulling an apple off a branch (0)

Chopping down a tree

Getting firewood (1)
Wielding an axe (0)

Measuring a room for carpeting

Getting ready to remodel (1)
Using a yard stick (0)

Cleaning the house

Showing one's cleanliness (1)
Vacuuming the floor (0)

Painting a room

- Making the room look fresh (1)
- Applying brush strokes (0)

Paying the rent

- Maintaining a place to live (1)
- Writing a check (0)

Washing clothes

- Removing odors from clothes (1)
- Putting clothes into the machine (0)

Supplementary Material E. Demographic Variables

Table S1. Demographic Variables.

Variables	Items	Study 1 (N = 240)	Follow-up of Study 1 (N = 234)	Study 2 N = 206	Follow-up A of Study 2 (N = 200)	Follow-up B of Study 2 (N = 211)	Follow-up C of Study 2 (N = 200)	Study 3 (N = 314)	Pre-test in Study 1 and Study 3 (N = 120)
Gender (%)	Male	42.9	42.3	41.3	43.5	39.8	42.5	48.4	44.2
	female	57.1	57.7	58.7	56.5	60.2	57.5	51.6	55.8
Age (%)	≤20	6.7	3.8	6.8	10.5	10.4	8	4.5	8.3
	21–30	55	50.4	53.9	45.5	54.5	51	46.2	51.7
	31–40	27.5	33.3	33	36.5	28	30	43.3	25.8
	41–50	5.4	6.8	2.9	5	3.8	6	4.8	8.3
	51–60	4.2	4.3	2.9	2.5	2.8	5	0.6	5.8
	>60	1.3	1.3	0.5	0	0.5	0	0.6	0
